# BreastSubtypeR: a unified R/Bioconductor package for intrinsic molecular subtyping in breast cancer research

**DOI:** 10.1093/nargab/lqaf131

**Published:** 2025-10-07

**Authors:** Qiao Yang, Johan Hartman, Emmanouil G Sifakis

**Affiliations:** Department of Oncology-Pathology, Karolinska Institutet, Stockholm 171 64, Sweden; Department of Oncology-Pathology, Karolinska Institutet, Stockholm 171 64, Sweden; Department of Clinical Pathology and Cancer Diagnostics, Karolinska University Hospital, Stockholm 171 76, Sweden; Department of Oncology-Pathology, Karolinska Institutet, Stockholm 171 64, Sweden

## Abstract

Summary: Intrinsic molecular subtyping (IS) of breast cancer is fundamental for understanding disease biology and guiding personalized treatment. While IS methods are standardized in clinical practice, their research implementations vary, leading to inconsistencies and reduced reproducibility. We introduce BreastSubtypeR, an R/Bioconductor package that unifies multiple published IS classifiers into a single, reproducible framework, enabling systematic cross-method comparison and robust cohort-aware selection. Its core feature, AUTO mode, quantitatively evaluates cohort composition and selectively activates classifiers whose assumptions are met, reducing user bias and improving robustness across skewed or subtype-specific cohorts. The package also provides a unified, method-specific normalization pipeline, optimized probe-to-gene mapping, and an intuitive local Shiny app (iBreastSubtypeR) for non-programmers. By standardizing method selection, preprocessing, and mapping within a Bioconductor workflow, BreastSubtypeR improves reproducibility and accessibility, and addresses an urgent need for assumption-aware IS in breast cancer research. Availability and implementation: The BreastSubtypeR package and its companion R Shiny application, iBreastSubtypeR, are freely available through Bioconductor (https://doi.org/10.18129/B9.bioc.BreastSubtypeR). The complete source code is also hosted on GitHub (https://github.com/JohanHartmanGroupBioteam/BreastSubtypeR). A citable archived snapshot of the version used (v1.1.3) is available on Zenodo (https://doi.org/10.5281/zenodo.17085316). The software is released under the GPL-3 license. Comprehensive documentation and an example dataset are provided to facilitate user adoption.

## Introduction

Breast cancer (BC) is a highly heterogeneous disease characterized by distinct intrinsic molecular subtypes with unique clinical, biological, and prognostic profiles [1–4]. Intrinsic molecular subtyping (IS) has become a cornerstone of BC research and clinical practice, enabling personalized treatment strategies and prognostic assessment. While assays like Prosigna^®^ have standardized IS for clinical use [5, 6], research applications remain fragmented due to variations in computational methods and underlying assumptions [7].

Recent methodological advances have sought to address these challenges from different angles. Post-GWAS (Genome-Wide Association Study) analyses, including Mendelian randomization [8] and fine-mapping platforms [9, 10], have revealed subtype-specific risk loci but do not directly provide gene-expression-based subtype labels. More recently, deep-learning and machine-learning approaches—such as autoencoder-based classifiers for bulk RNA-seq, integrative single-cell frameworks, image-based convolutional networks, and cascade models like BC-Predict—have demonstrated proof-of-concept for subtype prediction and prognosis [11–14]. Some of these models generate surrogate or cohort-specific labels (e.g. Luminal, HER2, TNBC), whereas others attempt to reproduce canonical intrinsic molecular subtypes. Nevertheless, traditional gene-expression-based methods such as PAM50 [15] remain the clinical and research standard for most applications.

Current, widely adopted methods, including PAM50 [15] and AIMS [16], classify tumours into the canonical intrinsic molecular subtypes: Luminal A, Luminal B, HER2-enriched, Basal-like, and Normal-like. However, challenges with these methods can vary in their application and often have limited adaptability to diverse datasets [7, 17]. These discrepancies often lead to reduced reproducibility and inconsistencies in IS results. Furthermore, while new subtypes are emerging in research, the canonical intrinsic molecular subtypes remain the most clinically relevant and widely adopted.

Existing IS tools have limitations. For example, the genefu package [18] on R/Bioconductor supports only a limited set of PAM50 variations and AIMS, restricting its applicability. The high-performing subgroup-specific gene-centering method (ssBC) [19, 20] is available solely as standalone R scripts, which limits accessibility for non-specialists. Moreover, to our knowledge, the two published immunohistochemistry (IHC)-based strategies [21, 22] are not publicly available, which makes adoption by researchers without computational expertise difficult. Together, these issues—limited method coverage, fragmented implementations, and restricted accessibility—impede routine cross-method benchmarking and reproducible adoption in BC research.

This absence of standardized, assumption-aware IS solutions therefore presents an urgent barrier to reproducible research. To address this gap, we developed BreastSubtypeR, a unified R/Bioconductor package that consolidates multiple IS methods into a single, user-friendly framework. Its key innovation, AUTO mode, quantitatively evaluates cohort composition (e.g. estrogen receptor (ER)/human epidermal growth factor receptor 2 (HER2) prevalence, subtype purity, subgroup size) and selectively enables only classifiers whose assumptions are satisfied, preventing misuse of nearest centroid (NC)-based methods in distributionally incompatible cohorts. In addition, BreastSubtypeR supports direct cross-method comparison (NC- and single-sample predictor (SSP)-based classifiers), method-specific normalization, optimized probe-to-gene mapping, and a local Shiny interface (iBreastSubtypeR) to broaden accessibility and ensure reproducibility.

By bridging methodological gaps and providing a versatile platform for IS, BreastSubtypeR empowers researchers to perform robust, assumption-aware, and reproducible analyses, advancing both basic and translational BC research. The package is available under a GPLv3 license via Bioconductor (https://doi.org/10.18129/B9.bioc.BreastSubtypeR), with comprehensive vignettes illustrating end-to-end workflows. Clinical validation against Prosigna^®^—via paired Prosigna^®^/RNA-seq datasets—will be pursued in future work.

## Materials and methods

### Multi-method subtyping: one-step analysis

BreastSubtypeR integrates ten IS methods, including both the original methods and their variations (Supplementary Table S1), providing a comprehensive toolkit for IS in breast cancer research. These methods include NC-based approaches like the original PAM50 [15] and ssBC [19, 20], as well as SSP-based methods such as AIMS [16] and the recently introduced sspbc models [23].

At the core of BreastSubtypeR lies the *BS_Multi* function, which enables simultaneous classifications using multiple IS methods. This functionality facilitates comparative analyses and explores the consistency between methods. The package calculates Shannon entropy to assess inter-method concordance, where lower entropy values indicate greater consistency among the selected methods. Details are provided in the Supplementary Material.

### AUTO mode feature

**Figure 1.**
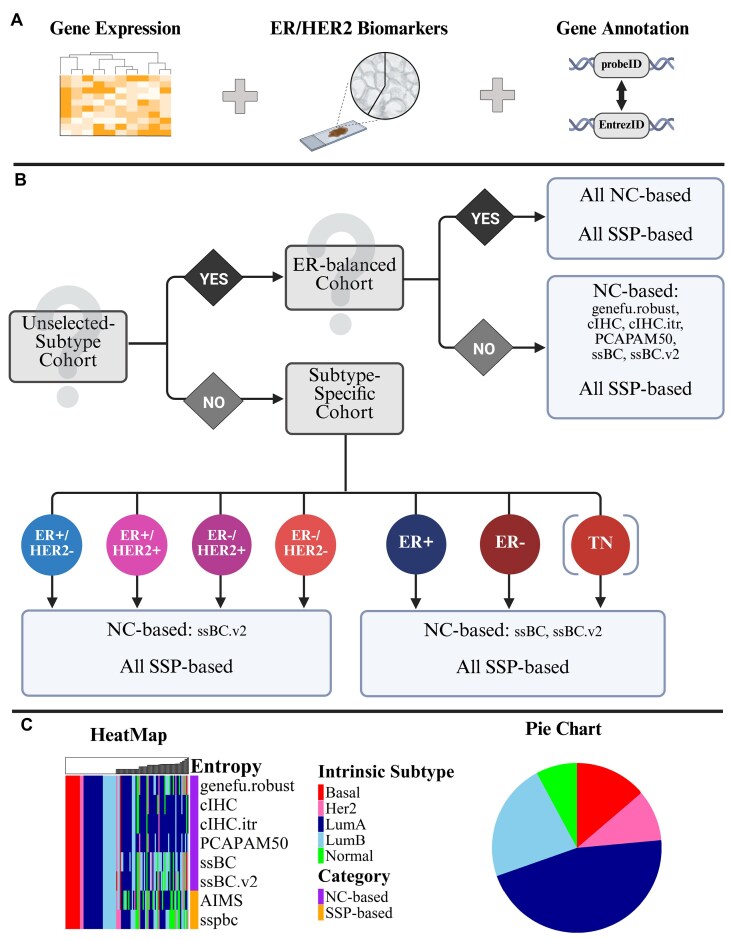
Overview of the *BS_Multi* function with AUTO mode enabled in the BreastSubtypeR package. (**A**) Required inputs for AUTO mode, including gene expression data, clinical biomarkers, and gene annotation data. Gene annotation is required for optimized gene mapping when using the *BS_Multi* function but is optional when running single-method IS. (**B**) Workflow of the method selection process within AUTO mode. The final selection of the most appropriate methods depends on the ER/HER2 distribution of the clinical samples. ER-balanced: The distribution of ER+/ER− samples is similar to that of the PAM50 training cohort (UNC232). Supported subtype-specific cohorts: ER+, ER−, ER+/HER2−, HER2+ (ER+/HER2+ and ER−/HER2+), and ER−/HER2−. For triple-negative (TN) BC cohorts, users must explicitly designate IHC-defined TN status to invoke the dedicated TNBC branch. NC-based: nearest centroid-based. SSP-based: single-sample predictor-based, including AIMS and sspbc methods. A detailed description of specific methods is provided in the Supplementary Material. (**C**) Example visualization output from BreastSubtypeR, showing IS analysis results using the package’s built-in plotting functions. LumA: Luminal A, LumB: Luminal B, Her2: HER2-enriched, Basal: Basal-like, and Normal: Normal-like. Created in BioRender. Sifakis, E. (2025) https://BioRender.com/cxb8lfk.

BreastSubtypeR introduces an innovative AUTO mode, which dynamically selects the most appropriate IS methods based on cohort-specific characteristics (Fig. 1 and Supplementary Fig. S8). The detailed logic behind the AUTO mode is described in Section 5 of the Supplementary Material. Datasets often exhibit subtype imbalances that violate the assumptions of specific IS methods. AUTO mode addresses this by evaluating key biomarkers, such as ER and HER2, and selecting methods that are more suitable for the given cohort. For instance, in ER-positive cohorts, AUTO mode selects methods tailored to these specific cohorts, such as the ssBC approach [19]. Importantly, the *BS_Multi* function enables simultaneous execution of multiple published classifiers for cross-method comparison. AUTO selects methods compatible with cohort characteristics but does not aggregate outputs into a forced consensus; any consensus label must be derived explicitly by the user if required.

The principles behind AUTO mode are as follows:

- *Unselected-subtype cohorts*: In cohorts encompassing all clinical subtypes, AUTO mode evaluates whether the ER+ fraction has ‘significantly deviated’ from the PAM50 training cohort (UNC232) [15] (54/118 ≈ 45.8%) by simulating 100 subcohorts (*N* = 118) from the SCAN-B cohort and requiring both a mean drop ≥5 percentage points (pp) in Cohen’s kappa and a one-sided *t*-test *P* < .01 (Supplementary Material Section 5.2, Fig. S2, and Table S3). This analysis yields Cohen’s kappa-based cutoffs of ER+ ≤ 39 % or ≥ 69 %. When ER+ lies outside these bounds, methods such as the conventional immunohistochemistry-based approach [22] or the robust implementation of genefu [18] are selected to account for these differences.
- *Subtype-specific cohorts*: In cohorts enriched for a specific clinical subtype—ER+, ER−, ER+/HER2−, ER+/HER2+, ER−/HER2+, ER−/HER2−, or triple negative (TN)—AUTO mode excludes methods, such as conventional implementations of genefu [18], which assume a broader subtype or ER-balanced distributions. Instead, AUTO selects methods more appropriate for these contexts, such as the ssBC [19] and/or ssBC.v2 [20] NC-based methods (with precomputed quantiles) alongside all SSP-based approaches. For TN cohorts, users must explicitly specify IHC-defined TN status (ER−/PR−/HER2−). Additionally, IS methods are selectively applied based on sample group size: NC-based methods are disabled if the total number of ER+ or ER− (or TN) samples is below 15 or 18, respectively; ssBC.v2 method is disabled for subgroups with fewer than 8 ER+/HER2− or 8 ER+/HER2+, or fewer than 9 ER−/HER2+ or 9 ER−/HER2− samples.
- *SSP-based methods*: SSP-based methods (e.g. AIMS [16], sspbc [23]) classify each sample independently using within-sample, pairwise gene-expression rules (for instance, ‘gene A > gene B’), and thus require no cohort-wide centering or scaling. This independence from the cohort’s overall gene-expression distribution renders SSP-based classifiers inherently robust to extreme ER+ or subtype imbalances (Supplementary Material Section 6.3.4, Fig. S7, and Table S6). Consequently, SSP-based methods are always included in AUTO mode, regardless of cohort composition.

By optimizing IS method selection, AUTO mode minimizes violations of underlying assumptions, improving both accuracy and reproducibility.

### Data input and normalization pipeline

BreastSubtypeR supports RNA-seq and microarray/nCounter data by automatically applying tailored normalization workflows to meet the input requirements of each method.

- Raw RNA-seq counts (with gene lengths)
  - *NC-based methods*: log_2_ counts per million (CPM) normalized via the upper-quartile method.
  - *SSP-based methods*: Linear fragments per kilobase of transcript per million mapped reads (FPKM), without cohort-level scaling.
- Precomputed RNA-seq FPKM (log_2_-transformed). When users supply log_2_-transformed FPKM, BreastSubtypeR:
  - *NC-based methods*: Ingest the matrix directly.
  - *SSP-based methods*: Back-transform to the linear scale (2^×^) before IS.
- Microarray or nCounter expression. We assume input is already log_2_-transformed, background-corrected, and normalized (e.g. RMA-processed microarray data).
  - *NC-based methods*: Ingest the normalized matrix as-is.
  - *SSP-based methods*: Back-transform to the linear scale (2^×^) before IS.

By consolidating raw and pre-normalized workflows, AUTO mode eliminates the need for manual preprocessing—users provide one gene expression matrix (plus gene lengths, if starting from raw counts), and BreastSubtypeR handles all downstream formatting. The complete pseudocode and parameter settings are provided in Section 7.1 of the Supplementary Material.

### Optimized gene mapping

Accurate gene mapping is a crucial step in IS, particularly when working with heterogeneous datasets or different platforms (e.g. microarrays versus RNA-seq). BreastSubtypeR incorporates an optimized gene-mapping strategy based on Entrez IDs. The mapping function verifies input datasets, identifies missing genes, and optimizes gene representation. This process minimizes discrepancies and ensures consistent results, thereby enhancing the reliability of subtyping analyses.

### User-friendly Shiny app interface

BreastSubtypeR includes the iBreastSubtypeR Shiny app, designed to allow users with minimal programming experience to perform single-method IS analyses (Supplementary Video 1 and Supplementary Fig. S9). The app runs locally, preserving data privacy and making it suitable for clinical applications. This intuitive graphic interface expands the accessibility of BreastSubtypeR, enabling a broader range of researchers to adopt its implemented IS methods.

### Wrapper fidelity check

To ensure that BreastSubtypeR’s wrapper faithfully reproduces each original algorithm, we benchmarked every implementation against its reference output using the SCAN-B cohort (*N* = 4606). We adopted the same default parameters and preprocessing procedures specified in each source publication whenever possible. For methods available as R packages or with publicly accessible code, we directly compared our wrapper’s subtype calls to those produced by the original implementation. Agreement was quantified using Cohen’s kappa via the psych v2.5.3 R package [24]. All IS methods included in BreastSubtypeR are catalogued in Table S1, with their corresponding package functions listed in Table S2.

### Comparative performance

To benchmark the performance of AUTO against the IS methods it excludes (hereafter referred to as ‘Excluded’), we evaluated three independent cohorts: SCAN-B, ABiM100, and OSLO2-EMIT0, across five real-world scenarios. In SCAN-B, we filtered the entire cohort to include only samples with available immunohistochemistry (IHC)-based subtype labels, alongside NCN-PAM50 calls, follow-up data, and surgical biopsy origin. This filtering yielded a final evaluation set of *N* = 4606 patients. From this SCAN-B subset, we generated 100 resampled subcohorts of five compositions: two skewed ER-positive mixes (ER+ ≈ 10% and ER+ ≈ 90%, *N* = 118) and three subtype-specific groups (pure ER+, pure ER−, pure TN). For ABiM100 and OSLO2-EMIT0, due to limited sample sizes, we assessed only the three subtype-specific subcohorts (pure ER+, pure ER−, pure TN) without resampling. For each scenario, we computed: (i) overall accuracy and Cohen’s kappa against NCN-PAM50 calls (SCAN-B only) and (ii) overall agreement with IHC-based subtypes (all cohorts; Supplementary Fig. S3). AUTO dynamically selects methods tailored to the cohort composition, whereas ‘Excluded’ represents all other available approaches. Full technical details are provided in Supplementary Sections 6.1 and 6.2.

## Limitations

While BreastSubtypeR addresses several key gaps in IS, there are limitations that should be considered:

- *Dependence on existing algorithms and lack of standardization in research settings*: BreastSubtypeR consolidates established IS methods, and while the use of AUTO mode helps improve accuracy by selecting the most suitable methods for a given cohort, the overall accuracy still depends on the inherent limitations of the research-based IS methods. BreastSubtypeR has not yet been validated against the Prosigna^®^ clinical assay. Future work will include direct Prosigna^®^ comparisons—e.g. via paired Prosigna^®^/RNA-seq samples—to quantify Cohen’s kappa and other agreement metrics. Importantly, BreastSubtypeR does not implement automatic consensus voting; AUTO performs assumption-aware method selection, while any consensus derivation remains user-defined.
- *Platform-specific performance variability*: The performance of IS methods may vary depending on the platform used. For example, while methods like the original PAM50 and its derivatives perform well with RNA-seq data, other methods (e.g. sspbc) may not perform as well with platforms such as microarrays or nCounter, which can impact the reproducibility of results.
- *Normalization defaults and lack of community consensus*: In the absence of a community-wide standard, we have adopted developer-recommended defaults for RNA-seq inputs: log_2_ upper-quartile CPM for NC-based methods [20, 25] and FPKM for SSP-based approaches [16]. We recognize that other strategies may be equally valid. Future BreastSubtypeR releases will incorporate configurable normalization options as head-to-head benchmarking studies become available.

To maintain alignment with emerging research needs, future versions of BreastSubtypeR will focus on:

- expanding method coverage by integrating novel algorithms for bulk transcriptomic data and performing harmonized benchmarking against emerging machine-learning and deep-learning models, using standardized preprocessing and shared gold-standard labels to enable fair, reproducible comparisons;
- incorporating IS methods for specific clinical subgroups, such as TN [26–28] and HER2-positive BC [29];
- extending functionality to single-cell transcriptomic data by including methods such as SCSubtype [30].

These directions aim to ensure that BreastSubtypeR remains not only a comprehensive repository of published classifiers but also a platform that evolves alongside methodological advances in IS for breast cancer research.

## Results

### Wrapper fidelity

Supplementary Fig. S1 demonstrates the fidelity of the BreastSubtypeR wrapper implementations by comparing their outputs to those of the original subtyping methods in the SCAN-B cohort. The agreement was assessed using Cohen’s kappa, with all comparisons yielding a value of 1.00, indicating perfect concordance. These findings confirm that the wrapper functions in BreastSubtypeR faithfully replicate the original algorithms, ensuring reproducibility and consistency in intrinsic molecular subtype assignments.

### Comparative performance

**Figure 2.**
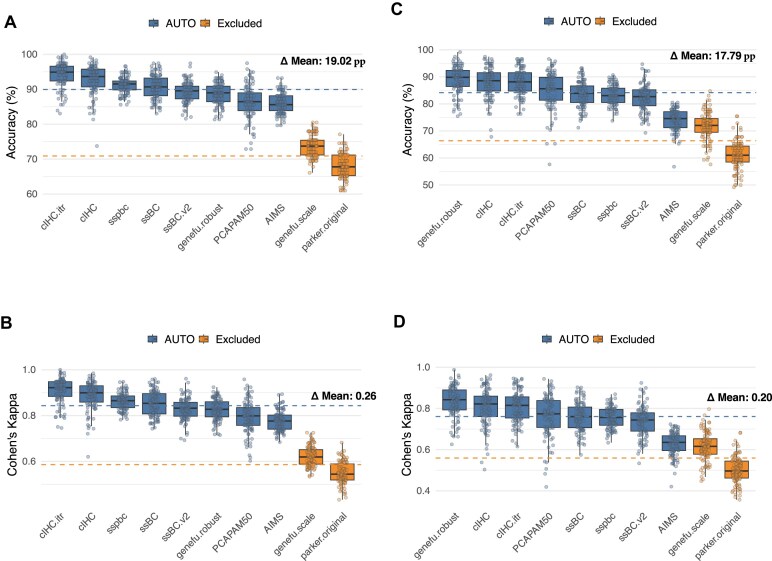
Performance of AUTO versus Excluded methods in SCAN-B subcohorts with skewed ER+ prevalence. Boxplots compare AUTO (blue) and Excluded classifiers (orange) across 100 resamples (*N* = 118) for: (**A**) overall accuracy in low-ER+ subcohorts (S10; ER+ ≈ 10%), (**B**) Cohen’s kappa in S10, (**C**) overall accuracy in high-ER+ subcohorts (S90; ER+ ≈ 90%), and (**D**) Cohen’s kappa in S90. Horizontal dashed lines indicate the overall mean for each group [blue = mean (AUTO), orange = mean (Excluded)]. The Δ label in each panel denotes the difference between these two means. pp: percentage points.

AUTO consistently outperformed the Excluded methods in head-to-head benchmarking across five distinct cohort scenarios. In SCAN-B simulations with skewed ER+ prevalence, AUTO demonstrated robust performance in both extremes. In low-ER+ subcohorts (S10; ER+ ≈ 10 %), AUTO achieved a mean accuracy of 89.90 ± 0.16 %, compared to 70.91 ± 0.31% for Excluded (Δ = +19.02 pp), and a Cohen's kappa of 0.84 ± 0.0024 versus 0.59 ± 0.0041 (Δ = +0.26) (Fig. 2A and B; Supplementary Table S4). In high-ER+ subcohorts (S90; ER+ ≈ 90%), AUTO maintained its advantage, with a mean accuracy of 84.15 ± 0.24% versus 66.36 ± 0.53% for Excluded (Δ = +17.79 pp), and a Cohen’s kappa of 0.76 ± 0.0034 compared to 0.56 ± 0.0062 (Δ = +0.20) (Fig. 2C and D; Supplementary Table S4). These improvements highlight AUTO’s ability to exclude centroid-based classifiers whose assumptions are violated in highly skewed datasets.

AUTO again demonstrated superior performance in subtype-specific cohorts (pure ER+, pure ER−, and pure TN) from SCAN-B, ABiM100, and OSLO2-EMIT0. In SCAN-B, accuracy against NCN-PAM50 increased by 24.58–31.61 pp, and Cohen’s kappa improved by 0.26–0.44 compared to Excluded (Supplementary Fig. S4 and Supplementary Table S5). In ABiM100 and OSLO2-EMIT0, agreement with IHC-based subtypes rose by 13.62–36.31 pp under AUTO (Supplementary Fig. S5 and Supplementary Table S6).

Moreover, we observed a strong inverse relationship between inter-classifier discordance—measured by Shannon entropy—and accuracy (Supplementary Fig. S6). AUTO’s cohort-aware selection not only boosts concordance with reference subtyping but also keeps entropy low, indicating more consistent predictions across classifiers.

Collectively, these results confirm that dynamic, cohort-aware method selection is essential for reliable intrinsic molecular subtyping, and that the classifiers excluded by AUTO would degrade performance if applied indiscriminately.

## Conclusions

BreastSubtypeR addresses critical challenges in BC research by providing a unified, multi-method platform for intrinsic molecular subtyping. By focusing on clinically relevant intrinsic molecular subtypes—Luminal A, Luminal B, HER2-enriched, and Basal-like—the package aligns with predominant translational research practices while maintaining flexibility to incorporate emerging methodologies. Its core innovations include:

- *Unified multi-method framework and cross-method comparison*: Ten published classifiers (both NC- and SSP-based) are harmonized under a single interface, enabling systematic benchmarking without ad hoc preprocessing.
- *Cohort-aware AUTO mode (not a consensus voter)*: Automatically evaluates cohort composition (e.g. ER/HER2 prevalence, subtype purity, subgroup size) and selectively disables incompatible classifiers, improving classification accuracy (up to +31.6 pp), Cohen’s kappa (up to +0.44), and IHC concordance (up to +31.6 pp).
- *Standardized input handling and method-specific normalization*: Supports raw RNA-seq, log_2_-FPKM, and log_2_-normalized microarray/nCounter data, ensuring reproducibility across platforms.
- *Optimized gene mapping across platforms*: Entrez-ID–based mapping reduces missing-marker bias and improves fidelity across technologies.
- *Dual accessibility and reproducibility*: A Shiny GUI (iBreastSubtypeR) broadens use for non-programmers, while a Bioconductor-compliant R API supports reproducible pipelines in translational research.

By consolidating heterogeneous implementations, enforcing assumption-aware method selection, and broadening accessibility, BreastSubtypeR fills a pressing need for reproducible and transparent intrinsic molecular subtyping in BC research. The package is freely available under a GPL-3 license via Bioconductor (https://doi.org/10.18129/B9.bioc.BreastSubtypeR), with comprehensive vignettes supporting end-to-end analysis.

## Supplementary Material

lqaf131_Supplemental_Files

## References

[1] Harbeck N, Penault-Llorca F, Cortes J et al. Breast cancer. Nat Rev Dis Primers. 2019; 5:66 10.1038/s41572-019-0111-2. 31548545

[2] Koboldt DC, Fulton RS, McLellan MD et al. Comprehensive molecular portraits of human breast tumours. Nature. 2012; 490:61–70. 10.1038/nature11412. 23000897 PMC3465532

[3] Sørlie T, Perou CM, Tibshirani R et al. Gene expression patterns of breast carcinomas distinguish tumor subclasses with clinical implications. Proc Natl Acad Sci USA. 2001; 98:10869. 11553815 10.1073/pnas.191367098 PMC58566

[4] Perou CM, Sørlie T, Eisen MB et al. Molecular portraits of human breast tumours. Nature. 2000; 406:747–52. 10.1038/35021093. 10963602

[5] Wallden B, Storhoff J, Nielsen T et al. Development and verification of the PAM50-based Prosigna breast cancer gene signature assay. BMC Med Genomics. 2015; 8:54 10.1186/s12920-015-0129-6. 26297356 PMC4546262

[6] Nielsen T, Wallden B, Schaper C et al. Analytical validation of the PAM50-based Prosigna Breast Cancer Prognostic Gene Signature Assay and nCounter Analysis System using formalin-fixed paraffin-embedded breast tumor specimens. BMC Cancer. 2014; 14:177 10.1186/1471-2407-14-177. 24625003 PMC4008304

[7] Prat A, Parker JS Standardized versus research-based PAM50 intrinsic subtyping of breast cancer. Clin Transl Oncol. 2020; 22:953 10.1007/s12094-019-02203-x. 31435878

[8] Tang SN, Zuber V, Tsilidis KK Identifying and ranking causal biochemical biomarkers for breast cancer: a Mendelian randomisation study. BMC Med. 2022; 20:457 10.1186/s12916-022-02660-2. 36424572 PMC9685978

[9] Wang T, Yan Z, Zhang Y et al. postGWAS: a web server for deciphering the causality post the genome-wide association studies. Comput Biol Med. 2024; 171:108108 10.1016/j.compbiomed.2024.108108. 38359659

[10] Watanabe K, Taskesen E, Van Bochoven A et al. Functional mapping and annotation of genetic associations with FUMA. Nat Commun. 2017; 8:1826 10.1038/s41467-017-01261-5. 29184056 PMC5705698

[11] Wang Y, Sun W, Karlsson E et al. Clinical evaluation of deep learning-based risk profiling in breast cancer histopathology and comparison to an established multigene assay. Breast Cancer Res Treat. 2024; 206:163–75. 10.1007/s10549-024-07303-z. 38592541 PMC11182789

[12] Gao F, Wang W, Tan M et al. DeepCC: a novel deep learning-based framework for cancer molecular subtype classification. Oncogenesis. 2019; 8:44 10.1038/s41389-019-0157-8. 31420533 PMC6697729

[13] Wang T, Mai D, Shu H et al. Enhancing cell subpopulation discovery in cancer by integrating single-cell transcriptome and expressed variants. Fundam Res. 2025; 10.1016/j.fmre.2025.01.001. PMC12869734 41647537

[14] Muthamilselvan S, Vaithilingam N, Palaniappan A BC-predict: mining of signal biomarkers and production of models for early-stage breast cancer subtyping and prognosis. Front Bioinform. 2025; 5:10.3389/fbinf.2025.1644695. PMC12488574 41048341

[15] Parker JS, Mullins M, Cheang MCU et al. Supervised risk predictor of breast cancer based on intrinsic subtypes. J Clin Oncol. 2009; 27:1160–7. 10.1200/JCO.2008.18.1370. 19204204 PMC2667820

[16] Paquet ER, Hallett MT Absolute assignment of breast cancer intrinsic molecular subtype. J Natl Cancer Inst. 2015; 107:dju357 10.1093/jnci/dju357. 25479802

[17] Staaf J, Ringnér M Making breast cancer molecular subtypes robust?. J Natl Cancer Inst. 2014; 107:dju386 10.1093/jnci/dju386. 25479803 PMC4301705

[18] Gendoo DMA, Ratanasirigulchai N, Schröder MS et al. Genefu: an R/Bioconductor package for computation of gene expression-based signatures in breast cancer. Bioinformatics. 2016; 32:1097 10.1093/bioinformatics/btv693. 26607490 PMC6410906

[19] Zhao X, Rødland EA, Tibshirani R et al. Molecular subtyping for clinically defined breast cancer subgroups. Breast Cancer Res. 2015; 17:29 10.1186/s13058-015-0520-4. 25849221 PMC4365540

[20] Fernandez-Martinez A, Krop IE, Hillman DW et al. Survival, pathologic response, and genomics in CALGB 40601 (Alliance), a neoadjuvant Phase III trial of paclitaxel-trastuzumab with or without lapatinib in HER2-positive breast cancer. J Clin Oncol. 2020; 38:4184–93. 10.1200/JCO.20.01276. 33095682 PMC7723687

[21] Curtis C, Shah SP, Chin SF et al. The genomic and transcriptomic architecture of 2,000 breast tumours reveals novel subgroups. Nature. 2012; 486:346–52. 10.1038/nature10983. 22522925 PMC3440846

[22] Ciriello G, Gatza ML, Beck AH et al. Comprehensive molecular portraits of invasive lobular breast cancer. Cell. 2015; 163:506–19. 10.1016/j.cell.2015.09.033. 26451490 PMC4603750

[23] Staaf J, Häkkinen J, Hegardt C et al. RNA sequencing-based single sample predictors of molecular subtype and risk of recurrence for clinical assessment of early-stage breast cancer. NPJ Breast Cancer. 2022; 8:94 10.1038/s41523-022-00465-3. 35974007 PMC9381586

[24] Revelle W Package “psych”—procedures for psychological, psychometric and personality research. R Package version 2.5.3. 2015; https://CRAN.R-project.org/package=psych.

[25] Fernandez-Martinez A, Rediti M, Tang G et al. Tumor intrinsic subtypes and gene expression signatures in early-stage ERBB2/HER2-positive breast cancer: a pooled analysis of CALGB 40601, NeoALTTO, and NSABP B-41 trials. JAMA Oncol. 2024; 10:603–11. 10.1001/jamaoncol.2023.7304. 38546612 PMC10979363

[26] Lehmann BD, Jovanović B, Chen X et al. Refinement of triple-negative breast cancer molecular subtypes: implications for neoadjuvant chemotherapy selection. PLoS One. 2016; 11:e0157368 10.1371/journal.pone.0157368. 27310713 PMC4911051

[27] Burstein MD, Tsimelzon A, Poage GM et al. Comprehensive genomic analysis identifies novel subtypes and targets of triple-negative breast cancer. Clin Cancer Res. 2015; 21:1688–98. 10.1158/1078-0432.CCR-14-0432. 25208879 PMC4362882

[28] Lehmann BD, Bauer JA, Chen X et al. Identification of human triple-negative breast cancer subtypes and preclinical models for selection of targeted therapies. J Clin Invest. 2011; 121:2750–67. 10.1172/JCI45014. 21633166 PMC3127435

[29] Rediti M, Venet D, Joaquin Garcia A et al. Identification of HER2-positive breast cancer molecular subtypes with potential clinical implications in the ALTTO clinical trial. Nat Commun. 2024; 15:10402 10.1038/s41467-024-54621-3. 39613746 PMC11607438

[30] Wu SZ, Al-Eryani G, Roden DL et al. A single-cell and spatially resolved atlas of human breast cancers. Nat Genet. 2021; 53:1334–47. 10.1038/s41588-021-00911-1. 34493872 PMC9044823

