# Supplementary material for: BreastSubtypeR: a unified R/Bioconductor package for intrinsic molecular subtyping in breast cancer research
Source: NAR Genom Bioinform. 2025 Oct 7;7(4):lqaf131. doi: 10.1093/nargab/lqaf131 (PMC12501779; doi:10.1093/nargab/lqaf131)
Supplement: lqaf131_Supplemental_Files [file lqaf131_supplemental_files.zip › BreastSubtypeR_ ApplicationNotes_Supplementary_revised_v7_CLEAN.pdf]

BreastSubtypeR: A Unified R/Bioconductor Package for Intrinsic Molecular Subtyping in Breast Cancer Research

Qiao Yang[1], Johan Hartman[1,2], and Emmanouil G. Sifakis[1]

1. Department of Oncology-Pathology, Karolinska Institutet, Stockholm, 171 64, Sweden
2. Department of Clinical Pathology and Cancer Diagnostics, Karolinska University Hospital, Stockholm, 171 76, Sweden

1 Main BreastSubtypeR Design Motivations

Breast cancer (BC) is a highly heterogeneous disease characterized by distinct molecular intrinsic subtypes (IS) with unique clinical, biological, and prognostic profiles (1–4). These subtypes—such as Luminal A, Luminal B, HER2-enriched, Basal-like, and Normal-like—are instrumental in guiding treatment strategies and prognostic assessment. While clinical assays like Prosigna® provide standardized subtyping for patient care (5,6), the research community still lacks consensus due to fragmented methods and difficulties adapting them across diverse datasets (7). This inconsistency undermines the reproducibility and reliability of scientific findings.

Current methods, such as the original PAM50 (8) and AIMS (9), have significantly advanced BC subtyping but suffer from challenges such as limited adaptability to varying datasets (7,10,11). These limitations often lead to difficulties in reproducing results across independent studies, especially when datasets come from different platforms or research environments. Furthermore, there is no centralized, accessible framework that integrates multiple subtyping methods with a focus on consistency and reliability.

Existing intrinsic subtyping tools, like the genefu package (12), are limited to a small subset of PAM50 variations and AIMS (9), restricting their use to a narrow range of studies. High-performing methods, such as subgroup-specific gene-centering (ssBC), perform well across various datasets but are not readily available as R packages (13,14); instead, they are distributed as standalone scripts. This makes it difficult for many researchers, particularly those without

advanced computational skills, to implement these methods. Additionally, traditional immunohistochemistry (IHC)-based strategies, such as the conventional ER-balancing using IHC approach (cIHC), remain inaccessible to most, limiting adoption without specialized expertise (15,16).

To address these challenges, BreastSubtypeR was developed as a comprehensive solution. This R/Bioconductor package integrates multiple molecular subtyping methods into a single, cohesive framework. By doing so, it allows researchers to conduct robust and reproducible subtyping analyses on BC datasets of various sizes and platforms. Including the AUTO mode enables the dynamic selection of the most appropriate methods based on the dataset's characteristics, improving adaptability and efficiency. Furthermore, BreastSubtypeR incorporates an optimized gene mapping strategy to overcome inconsistencies in gene sets, further enhancing reproducibility.

Importantly, BreastSubtypeR is designed to be a highly accessible tool. The package includes an interactive Shiny application (iBreastSubtypeR), offering a user-friendly graphic interface for both bioinformaticians and researchers with limited R programming experience. This makes subtyping analyses more accessible to researchers across diverse fields, from bioinformatics to clinical research, without requiring deep technical knowledge of the underlying methods. By bridging the gap between computational expertise and clinical application, BreastSubtypeR facilitates BC research and ultimately contributes to advancing our understanding of this complex disease.

2 Implemented Molecular Subtyping Approaches

BreastSubtypeR integrates several published molecular subtyping methods that classify tumors into widely recognized IS: Luminal A, Luminal B, HER2-enriched, Basal-like, and Normal-like, offering a comprehensive toolkit for BC subtyping. The package supports both five-subtype and four-subtype classifications (excluding the Normal-like subtype) to align with the standardized PAM50 clinical test.

Table S1 lists all molecular subtyping methods and their variations implemented in BreastSubtypeR as of August 2025. These include nearest centroid (NC)-based methods like the original PAM50 (8) and subgroup-specific gene-centering (ssBC) approach (13), as well as single-

sample predictor (SSP)-based methods like AIMS (9), and the recently published sspbc models (17).

Table S1. Intrinsic molecular subtype classification methods implemented in BreastSubtypeR as of February 2025.

Method	Category	IHC Input Requirement	Description	Citation
parker.original	NC-based	Not required	Original PAM50 by Parker *et al*. 2009	(8)
genefu.scale	NC-based	Not required	PAM50 implementation as in the genefu R package (scaled version)	(12)
genefu.robust	NC-based	Not required	PAM50 implementation as in the genefu R package (robust version)	(12)
cIHC	NC-based	IHC ER status	Conventional ER-balancing using immunohistochemistry (IHC)	(16)
cIHC.itr	NC-based	IHC ER status	Iterative version of cIHC	(15)
PCAPAM50	NC-based	IHC ER status → ESR1	Selects IHC-defined ER subsets, then uses Principal component analysis (PCA) to create ESR1 expression-based ER-balancing	(18)
ssBC	NC-based	IHC ER, or Triple Negative (TN) status	Subgroup-specific gene-centering PAM50	(13)
ssBC.v2	NC-based	IHC ER and HER2, or TN status	Updated subgroup-specific gene-centering PAM50 with refined quantiles	(14)
AIMS	SSP-based	Not required	Absolute Intrinsic Molecular Subtyping (AIMS) method	(9)
sspbc	SSP-based	Not required	Single-Sample Predictors for Breast Cancer (AIMS adaptation)	(17)

3 Wrapper Fidelity Check

Table S2 details every subtyping algorithm in BreastSubtypeR, showing each method's name, its corresponding R-wrapper function, and the availability of the original implementation. For methods distributed as R packages or with published R source code, we directly compared IS calls

from our wrapper against those from the reference implementation using the SCAN-B cohort (N = 4,606) – each comparison yielded Cohen's kappa = 1.0, confirming exact reproduction (Fig. S1). The ssBC.v2 method is provided only as Python source code, and its formal fidelity check is pending (14). The cIHC.itr method had no external implementation (listed as "N/A") (15) and was therefore validated for internal consistency and correct parameter handling.

Table S2. Implementation and validation details for subtyping methods in BreastSubtypeR. Each row lists the method name, the corresponding R-wrapper function, the source of the original implementation (R package, R source, Python source, or not available (N/A)), and the validation status of the wrapper.

Method	Wrapper Call	Original Availability	Validation Status
parker.original	BS_parker(calibration = "Internal", internal = "-1", ...)	R source code	Validated against original
genefu.scale	BS_parker(calibration = "Internal", internal = "meanCtr", ...)	R package (genefu)	Validated against package
genefu.robust	BS_parker(calibration = "Internal", internal = "qCtr", ...)	R package (genefu)	Validated against package
cIHC	BS_cIHC(...)	R package (PCAPAM50)	Validated against package
cIHC.itr	BS_cIHC.itr(...)	N/A	Parameter behavior checked
PCAPAM50	BS_PCAPAM50(...)	R package (PCAPAM50)	Validated against package
ssBC	BS_ssBC(s = "ER", ...)	R source code	Validated against original
ssBC.v2	BS_ssBC(s = "ER.v2", ...)	Python source code	Validation pending
AIMS	BS_AIMS(...)	R package (AIMS, genefu)	Validated against AIMS
sspbc	BS_sspbc(...)	R package (sspbc)	Validated against package

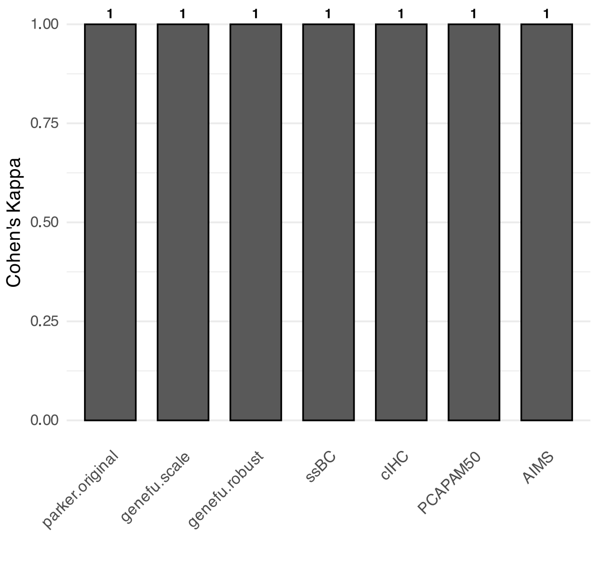

Figure S1. Agreement between BreastSubtypeR wrappers and their reference implementations, quantified by Cohen's kappa. Each bar represents one method with available R source or package code (Cohen's kappa = 1.0 for all), demonstrating exact replication of IS assignments. Methods without R references – ssBC.v2 (Python only) and cIHC.itr (no external code) – are not included in this comparison.

4 Concordance of Subtyping Methods Using Shannon Entropy

BreastSubtypeR calculates Shannon entropy, as a supplementary metric to quantify the variability in IS assignments across methods, providing insight into the reliability and agreement between different subtyping approaches. A lower entropy value indicates higher concordance among methods for a given sample, whereas a higher entropy value reflects greater variability and lower agreement. When running the *BS_Multi* function, Shannon entropy (H) is computed for each sample as follows:

$$H = -\sum_{i} P_i * \ log_2(P_i)$$

Where:

- P_i represents the probability (frequency) of intrinsic molecular subtype i for a given sample, derived from the classification results of all selected subtyping methods.
- The summation runs over all possible IS assigned to the sample.

By calculating Shannon entropy, BreastSubtypeR provides a quantitative measure of inter-method concordance. This entropy-based approach helps researchers evaluate the consistency of subtyping results and assess whether different methods yield comparable classifications across datasets.

5 Optimized Method Selection in AUTO mode

5.1 Logic in AUTO mode

The get_methods() function dynamically tailors the set of breast cancer subtyping methods to the input cohort's characteristics, as defined in the supplied phenotype table. First, if any triple-negative ("TN") column is present, TN-specific nearest-centroid (NC)-based methods (ssBC, ssBC.v2) are activated (13,14). Next, for HER2-enriched cohorts – identified by the absence of

both ER+/HER2- and ER-/HER2- samples – HER2-aware NC-based are prioritised, such as ssBC.v2 (14). The routine then inspects the ER and HER2 distributions using configurable minimum thresholds (default: ≥ 10 samples for overall ER status; ≥ 5 per ER/HER2 subtype) to detect ER-dominant or ER-depleted cohorts. The full suite of NC-based methods is enabled if the cohort's ER+ proportion falls within a balanced window (36-69%, see analysis in the next section). In all cases, single-sample predictors (SSP) remain available to ensure robust classification across heterogeneous datasets, including AIMS (9) and sspbc (17). This hierarchical logic ensures that AUTO mode selects only those methods best suited to each cohort's characteristics, maximizing accuracy while preserving flexibility. The R pseudocode for this decision tree is provided below.

```
FUNCTION get_methods(pheno):
  # Initialize defaults
  cohort.select = "ERpos"
  samples_ER.icd = NULL
  samples_ERHER2.icd = NULL
  methods = NULL

  # Case 1: No phenotype data provided
  IF pheno has no columns:
    WARN "No pheno table detected."
    SET methods = ["AIMS", "sspbc"]

  # Case 2: Missing required columns (ER/HER2)
  ELSE IF "ER" or "HER2" not in pheno columns:
    ERROR "AUTO mode requires 'ER' and 'HER2' columns."

  # Case 3: Phenotype data available
  ELSE:
    # Calculate sample counts for ER/HER2 subtypes
    n_ERpos = count ER+ samples
    n_ERneg = count ER- samples
    n_ERposHER2pos, n_ERposHER2neg, n_ERnegHER2pos, n_ERnegHER2neg = count respective subtypes

    # Define thresholds
    n_ERpos_threshold = 15
    n_ERneg_threshold = 18
    n_ERposHER2pos_threshold = round(n_ERpos_threshold / 2)
    n_ERposHER2neg_threshold = round(n_ERpos_threshold / 2)
    n_ERnegHER2pos_threshold = round(n_ERneg_threshold / 2)
    n_ERnegHER2neg_threshold = round(n_ERneg_threshold / 2)
    upper_ratio = 0.69
    lower_ratio = 0.36

    # Rule 1: TN cohort
    IF "TN" column exists in pheno:
      SET cohort.select = "TN"
      SET methods = ["ssBC", "ssBC.v2", "AIMS", "sspbc"]

    # Rule 2: HER2+ cohort
    ELSE IF no ER+/HER2- or ER-/HER2- samples:
      SET cohort.select = "HER2pos"
      IF HER2+ samples < threshold:
```

```
        SET methods = ["AIMS", "sspbc"]
    ELSE:
        SET methods = ["ssBC.v2", "AIMS", "sspbc"]

  # Rule 3: Small ER+/- cohort
  ELSE IF both ER+ and ER- samples < threshold:
      SET methods = ["AIMS", "sspbc"]

  # Rule 4: ER+ dominant cohort
  ELSE IF ER+ ≥ threshold AND ER- < threshold:
      IF ER+/HER2+ and ER+/HER2- both ≥ threshold:
          SET methods = ["ssBC", "ssBC.v2", "AIMS", "sspbc"]
      ELSE IF only one ER+/HER2 subtype ≥ threshold:
          SET methods = ["ssBC", "ssBC.v2", "AIMS", "sspbc"]

  # Rule 5: ER- dominant cohort
  ELSE IF ER- ≥ threshold AND ER+ < threshold:
      Similar logic as Rule 4, but for ER- subtypes

  # Rule 6: Balanced ER+/ER- cohort
  ELSE IF both ER+ and ER- ≥ threshold:
      ratio_ER = n_ERpos / ( n_ERpos  +  n_ERneg)
      IF ratio_ER within bounds (lower_ratio, upper_ratio):
          SET methods = ["parker.original", "genefu.scale", "genefu.robust", ..., "sspbc"]
      ELSE:
          SET methods = ["genefu.robust", "ssBC", "ssBC.v2", ..., "sspbc"]

  # Subset samples for ssBC/ssBC.v2 if needed
  IF cohort.select ≠ "TN":
      # Identify valid ER/HER2 subtypes with enough samples
      valid_ER_subtypes = [subtypes with counts > n_ER_threshold]
      valid_ERHER2_subtypes = [subtypes with counts > n_ERHER2_threshold]

      # Map patient IDs for ssBC (ER-based) and ssBC.v2 (ER/HER2-based)
      samples_ER.icd = get patient IDs for valid_ER_subtypes
      samples_ERHER2.icd = get patient IDs for valid_ERHER2_subtypes

RETURN:
  samples_ER.icd,
  samples_ERHER2.icd,
  methods,
  cohort.select
```

5.2 Data-Driven Determination of ER+ Skew Thresholds

We empirically derived ER+ fraction cutoffs that mark significant performance degradation by analyzing the SCAN-B cohort (N = 4,606). Specifically, we generated 100 random subcohorts (N = 118) at ER+ proportions from 10 % to 90 % in 1 percentage points (pp) increments, classified each replicate with the original Parker et al. (2009) PAM50 nearest-centroid method, and recorded overall accuracy and Cohen's kappa against published SCAN-B NCN.PAM50 labels. At each ER+ level, we compared the 100-replicate metrics to the baseline distribution at ≈ 45.8 % ER+ (the UNC232 original training cohort rate) using one-sided t-tests (alternative = "less"). We defined an

ER+ fraction as "significantly skewed" if the mean metric dropped by ≥ 5 percentage points (pp) relative to baseline and the t-test p-value was < 0.01. The resulting cutoffs – ≤ 36 % and ≥ 70 % ER+ for accuracy, and ≤ 39 % and ≥ 69 % ER+ for Cohen's kappa – are presented in Table S3 and visualized in Figure S2. Because kappa adjusts for chance agreement and is more sensitive to imbalance, we adopt the Cohen's kappa-based thresholds (39 %/69 % ER+) as the primary bounds in AUTO mode to detect and compensate for ER+ skew.

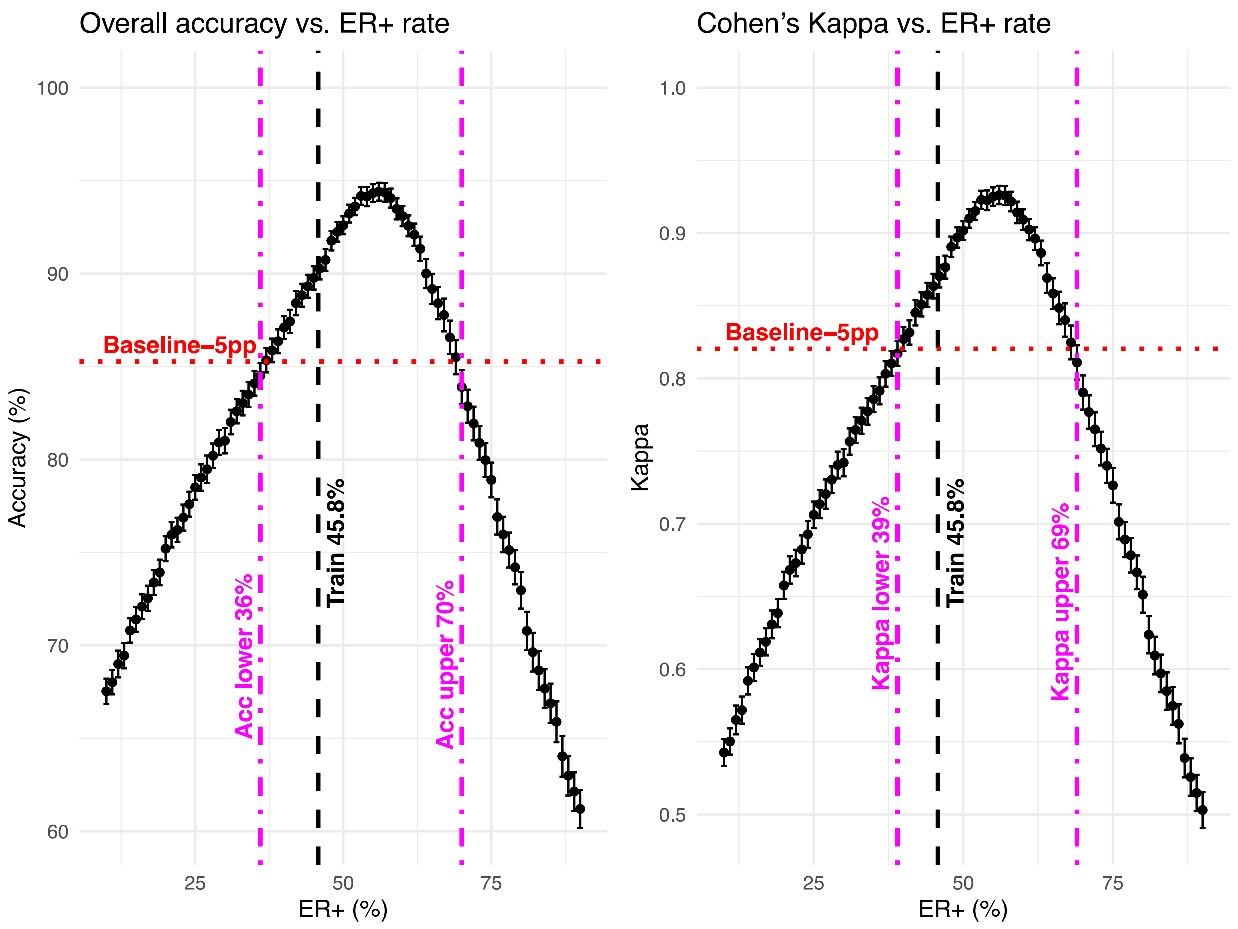

Figure S2. ER+ composition sensitivity analysis of PAM50 performance in SCAN-B subcohorts. Each panel shows the mean performance (± 95 % confidence interval (CI)) across 100 resampled subcohorts (N = 118) at varying ER+ proportions, using (left) overall accuracy and (right) Cohen's kappa against reference NCN-PAM50 calls and the original Parker et al. (2009) implementation (parker.original). The vertical dashed black line indicates the baseline ER+ rate (≈ 45.8%). Horizontal red dotted lines mark a ≥ 5 percentage point (pp) drop from baseline performance. Vertical magenta dashed/dotted lines denote ER+ thresholds where the decline was both statistically significant (p < 0.01) and ≥ 5 pp from baseline, defining the data-driven cutoffs for identifying skewed cohorts.

Table S3. Data-driven identification of ER+ composition cutoffs based on PAM50 performance in SCAN-B resampled subcohorts.

Subcohort (ER rate (%))	Accuracy (Mean ± SE)	Kappa (Mean ± SE)	p-value (Accuracy)	p-value (Kappa)
10%	67.5 ± 0.34%	0.54 ± 0.0046	1.57E-113	1.79E-118
11%	68.0 ± 0.33%	0.55 ± 0.0046	3.01E-114	5.93E-118
12%	69.0 ± 0.36%	0.57 ± 0.0050	1.91E-104	1.01E-107
13%	69.5 ± 0.34%	0.57 ± 0.0047	2.20E-106	2.28E-110
14%	70.8 ± 0.33%	0.59 ± 0.0047	1.36E-103	5.60E-105
15%	71.4 ± 0.34%	0.60 ± 0.0047	3.45E-100	1.19E-102
16%	72.1 ± 0.33%	0.61 ± 0.0046	4.52E-98	1.53E-100
17%	72.5 ± 0.34%	0.62 ± 0.0047	7.73E-95	3.83E-97
18%	73.4 ± 0.34%	0.63 ± 0.0048	2.66E-91	3.84E-92
19%	73.9 ± 0.35%	0.64 ± 0.0049	8.98E-88	9.55E-89
20%	75.2 ± 0.34%	0.66 ± 0.0047	4.05E-83	1.75E-84
21%	75.9 ± 0.34%	0.67 ± 0.0047	5.82E-79	5.75E-81
22%	76.2 ± 0.33%	0.67 ± 0.0046	1.52E-79	7.91E-81
23%	76.9 ± 0.35%	0.68 ± 0.0049	9.86E-73	4.88E-74
24%	77.6 ± 0.34%	0.69 ± 0.0047	5.23E-71	1.09E-72
25%	78.5 ± 0.34%	0.71 ± 0.0046	1.78E-66	5.57E-68
26%	79.0 ± 0.36%	0.71 ± 0.0049	1.41E-60	3.00E-62
27%	79.5 ± 0.37%	0.72 ± 0.0051	2.75E-56	1.73E-57
28%	80.2 ± 0.33%	0.73 ± 0.0046	4.29E-57	7.05E-58
29%	80.9 ± 0.34%	0.74 ± 0.0046	6.92E-52	5.26E-53
30%	81.0 ± 0.34%	0.74 ± 0.0047	9.89E-51	1.76E-51
31%	82.0 ± 0.33%	0.76 ± 0.0046	2.12E-45	3.73E-46
32%	82.6 ± 0.33%	0.76 ± 0.0045	4.93E-42	1.12E-42
33%	83.0 ± 0.33%	0.77 ± 0.0045	1.34E-38	2.59E-39
34%	83.5 ± 0.34%	0.78 ± 0.0046	6.40E-35	1.25E-35
35%	84.1 ± 0.33%	0.79 ± 0.0045	2.02E-31	4.54E-32
36%	84.5 ± 0.35%	0.79 ± 0.0047	1.60E-27	3.96E-28
37%	85.3 ± 0.33%	0.80 ± 0.0044	5.29E-23	1.70E-23
38%	85.9 ± 0.32%	0.81 ± 0.0043	5.73E-20	2.28E-20
39%	86.4 ± 0.32%	0.82 ± 0.0043	1.29E-16	5.21E-17
40%	87.1 ± 0.31%	0.83 ± 0.0041	1.35E-12	6.54E-13
41%	87.4 ± 0.31%	0.83 ± 0.0042	1.81E-10	9.52E-11
42%	88.4 ± 0.33%	0.85 ± 0.0044	1.86E-05	1.51E-05
43%	88.8 ± 0.31%	0.85 ± 0.0042	4.75E-04	4.25E-04
44%	89.3 ± 0.31%	0.86 ± 0.0042	1.37E-02	1.30E-02

45%	89.8 ± 0.32%	0.86 ± 0.0043	1.24E-01	1.19E-01
46%	90.3 ± 0.29%	0.87 ± 0.0039	-	-
47%	90.7 ± 0.30%	0.88 ± 0.0040	8.68E-01	8.64E-01
48%	91.8 ± 0.26%	0.89 ± 0.0035	1.00E+00	1.00E+00
49%	92.2 ± 0.26%	0.90 ± 0.0035	1.00E+00	1.00E+00
50%	92.6 ± 0.24%	0.90 ± 0.0032	1.00E+00	1.00E+00
51%	93.2 ± 0.24%	0.91 ± 0.0032	1.00E+00	1.00E+00
52%	93.6 ± 0.23%	0.92 ± 0.0031	1.00E+00	1.00E+00
53%	94.2 ± 0.24%	0.92 ± 0.0031	1.00E+00	1.00E+00
54%	94.1 ± 0.26%	0.92 ± 0.0034	1.00E+00	1.00E+00
55%	94.3 ± 0.24%	0.93 ± 0.0032	1.00E+00	1.00E+00
56%	94.4 ± 0.24%	0.93 ± 0.0032	1.00E+00	1.00E+00
57%	94.4 ± 0.25%	0.93 ± 0.0033	1.00E+00	1.00E+00
58%	94.1 ± 0.26%	0.92 ± 0.0034	1.00E+00	1.00E+00
59%	93.5 ± 0.29%	0.91 ± 0.0037	1.00E+00	1.00E+00
60%	93.1 ± 0.27%	0.91 ± 0.0036	1.00E+00	1.00E+00
61%	92.6 ± 0.28%	0.90 ± 0.0037	1.00E+00	1.00E+00
62%	92.1 ± 0.30%	0.90 ± 0.0039	1.00E+00	1.00E+00
63%	91.3 ± 0.33%	0.89 ± 0.0043	9.92E-01	9.97E-01
64%	90.0 ± 0.40%	0.87 ± 0.0051	2.95E-01	4.21E-01
65%	89.2 ± 0.41%	0.86 ± 0.0053	1.55E-02	3.51E-02
66%	88.4 ± 0.44%	0.85 ± 0.0056	2.55E-04	8.55E-04
67%	87.8 ± 0.44%	0.84 ± 0.0058	2.83E-06	1.25E-05
68%	86.6 ± 0.45%	0.82 ± 0.0059	6.79E-11	5.10E-10
69%	85.5 ± 0.46%	0.81 ± 0.0060	1.40E-15	1.31E-14
70%	83.9 ± 0.46%	0.79 ± 0.0059	1.04E-23	1.03E-22
71%	82.9 ± 0.45%	0.78 ± 0.0058	7.58E-30	8.31E-29
72%	81.9 ± 0.46%	0.76 ± 0.0059	3.23E-34	3.27E-33
73%	80.9 ± 0.46%	0.75 ± 0.0058	2.36E-39	1.26E-38
74%	80.0 ± 0.46%	0.74 ± 0.0059	1.33E-43	8.66E-43
75%	78.9 ± 0.47%	0.73 ± 0.0060	1.21E-47	3.93E-47
76%	76.9 ± 0.48%	0.70 ± 0.0061	1.23E-55	2.46E-55
77%	76.0 ± 0.48%	0.69 ± 0.0061	5.52E-59	7.31E-59
78%	75.1 ± 0.47%	0.68 ± 0.0060	3.87E-63	2.17E-63
79%	74.2 ± 0.46%	0.67 ± 0.0058	4.70E-68	1.62E-68
80%	73.0 ± 0.50%	0.65 ± 0.0062	6.32E-68	6.80E-69
81%	70.8 ± 0.52%	0.62 ± 0.0065	2.69E-72	8.69E-74
82%	69.6 ± 0.52%	0.61 ± 0.0065	1.05E-74	1.52E-76
83%	68.7 ± 0.53%	0.60 ± 0.0065	3.07E-77	2.03E-79
84%	67.7 ± 0.53%	0.58 ± 0.0065	5.71E-80	1.09E-82

85%	66.9 ± 0.53%	0.57 ± 0.0066	7.43E-81	6.50E-84
86%	65.9 ± 0.55%	0.56 ± 0.0068	8.05E-81	2.73E-84
87%	64.0 ± 0.55%	0.54 ± 0.0067	4.56E-85	1.29E-89
88%	63.0 ± 0.54%	0.53 ± 0.0065	7.09E-90	1.02E-94
89%	62.1 ± 0.52%	0.51 ± 0.0063	4.25E-94	2.47E-99
90%	61.2 ± 0.51%	0.50 ± 0.0062	1.18E-97	5.04E-103

6 Comparative Performance

6.1 Study Cohorts

We benchmarked the performance of AUTO mode against the subtyping methods it excludes (hereafter referred to as "Excluded") using three publicly available breast cancer cohorts: SCAN-B (17), ABiM (19), and OSLO2-EMIT0 (20,21), all retrieved from Mendeley Data (17).

The SCAN-B cohort comprised 4,606 samples that were rigorously filtered to include only surgical specimens (BiopsyType == "OP"), samples from the follow-up cohort (Follow.up.cohort == "TRUE"), with complete immunohistochemistry (IHC)-based subtype labels – defined as Triple-Negative (TN; ER-/PR-/HER2-), HER2-enriched (ER-/PR-/HER2+), Luminal A (ER+/HER2-/Ki67-), Luminal B1 (ER+/HER2-/Ki67+), and Luminal B2 (ER+/HER2+) – and NCN-PAM50 calls (22–24).

The ABiM100 (N = 99 after excluding one sample with incomplete biomarkers) and OSLO2-EMIT0 (N = 102 after excluding one patient with missing HER2 status) cohorts were used as additional validation datasets. Initially, these cohorts lacked complete Ki67 biomarker data and could not be directly assigned Luminal A vs. Luminal B1 clinical subtypes.

To enable consistent IHC-based subtyping across all cohorts, we followed a robust Ki67 status inference strategy (18) using the larger ABiM405 cohort, with consensus IHC biomarker annotations (25) as the reference. We first performed cross-cohort expression harmonization using the *sva* R package (26) to correct for batch effects. Next, we conducted density-based analyses of MKI67 expression in harmonized ABiM405 data aligned to each target cohort (ABiM100 and OSLO2-EMIT0). From these distributions, we derived bimodal expression thresholds that

distinguished Luminal A (LA) from Luminal B1 (LB1) tumors (Figure S3). These thresholds were then applied to infer Ki67 status in the ABiM100 and OSLO2-EMIT0 datasets. By integrating ER, PR, HER2, and inferred Ki67 status, we constructed complete IHC-based subtypes in all samples, ensuring comparability across all benchmarking analyses.

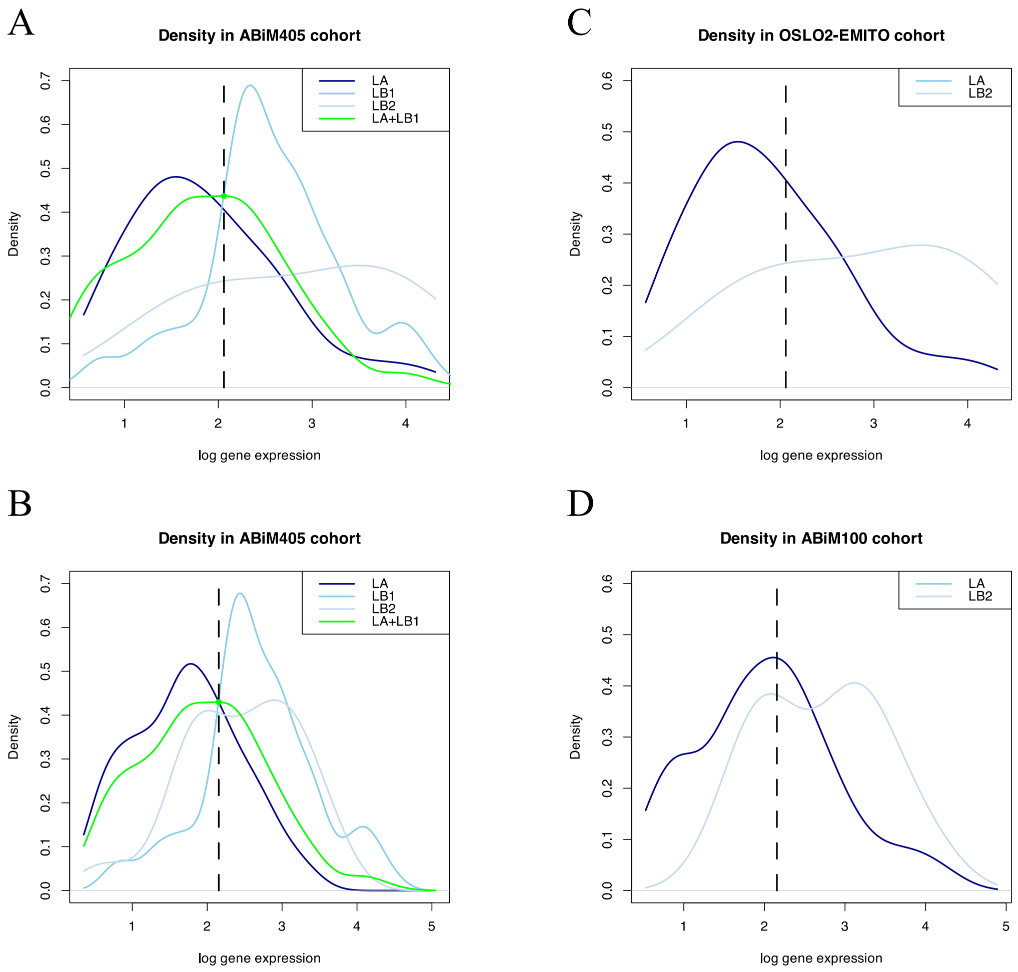

Figure S3. MKI67 expression threshold determination for Luminal subtyping. Density plots of batch-corrected MKI67 expression in ABiM405 aligned to (A) OSLO2-EMIT0 and (B) ABiM100. Vertical dashed lines indicate the LA/LB1 discrimination threshold. This threshold was applied to classify LA vs. LB1 subtypes in (C) OSLO2-EMIT0 and (D) ABiM100, enabling IHC-based subtyping.

6.2 Execution Details

6.2.1 Execution Modes

- **AUTO**: Runs a data-driven subset of classifiers selected according to cohort composition. Excludes approaches whose training assumptions (e.g., balanced ER+ distribution) do not match the cohort structure (e.g., skewed or pure TN).
- **Excluded**: Forces execution of all available classifiers omitted by AUTO for direct comparison.

6.2.2 Performance Metrics

- **SCAN-B:** Overall accuracy (%) and Cohen's kappa against NCN-PAM50 reference.
- **All cohorts:** Agreement (%) with IHC-based subtype calls.

6.2.3 Reference Annotations

- **SCAN-B:** NCN-PAM50 IS assignments.
- **All cohorts:** IHC-based subtype labels derived from ER/PR/HER2/Ki67 (inferred when missing).

6.2.4 Statistical Summary

- **SCAN-B:** Mean ± SE reported across 100 simulations for S10, S90, or pure subtype compositions.
- **All cohorts:** Delta **(**Δ = AUTO – Excluded) reported per metric.

6.3 Comparative Performance

6.3.1 SCAN-B

Table S4. Performance metrics (mean ± SE) comparing AUTO versus Excluded methods across 100 replicates (N = 118) in SCAN-B subcohorts with skewed ER+ prevalence: S10 (ER+ ≈ 10%) and S90 (ER+ ≈ 90%).

Subcohort	Method	Accuracy (Mean ± SE)	Kappa (Mean ± SE)
S10 (ER+ ≈ 10%)	AUTO	89.93 ± 0.16%	0.8432 ± 0.0024
	Excluded	70.91 ± 0.31%	0.5862 ± 0.0041
S90 (ER+ ≈ 90%)	AUTO	84.15 ± 0.24%	0.761 ± 0.0034
	Excluded	66.36 ± 0.53%	0.5592 ± 0.0062

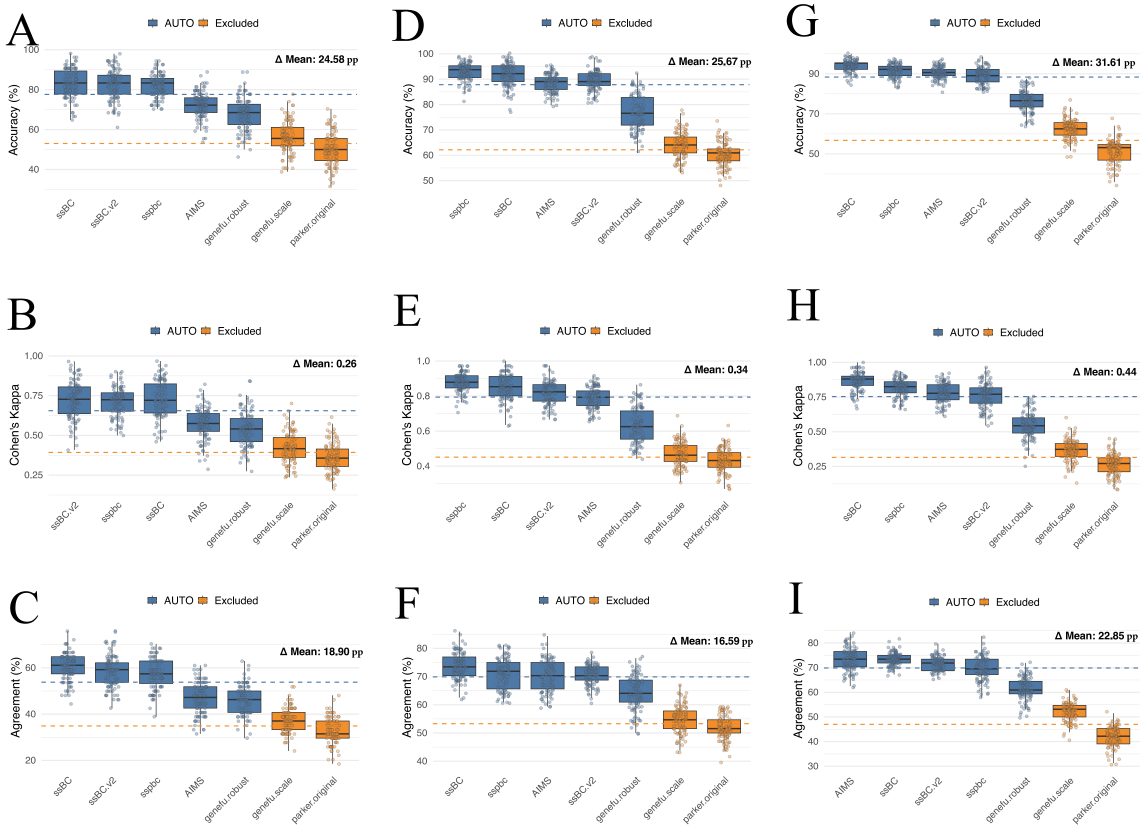

Figure S4. Performance comparison of AUTO (blue) versus Excluded approaches (orange) for subtyping pure ER+, ER-, and triple-negative (TN) breast tumors in the SCAN-B cohort. Boxplots show distributions across 100 iterations for ER+ samples (N = 54) in panels A-C, ER- samples (N = 54) in panels D-F, and TN samples (N = 64) in panels G-I. For each group, performance is evaluated by (A, D, G) overall accuracy against NCN-PAM50 IS, (B, E, H) Cohen's kappa against NCN-PAM50 IS, and (C, F, I) agreement rate with IHC-based subtypes. Horizontal dashed lines indicate group means, and the mean difference (Δ) between the AUTO and Excluded methods is annotated in each panel. pp: percentage points.

Table S5. Performance metrics comparing AUTO versus Excluded methods in pure ER+ (N = 54), pure ER- (N = 64), and pure triple-negative (TN; N = 64) subcohorts from the SCAN-B cohort. Metrics are reported as mean ± SE across 100 iterations (100i) for each group.

Subcohort (100i)	Reference	Method	Accuracy (Mean ± SE)	Kappa (Mean ± SE)	IHC Agreement (Mean ± SE)
ER+ (N = 54)	NCN-PAM50	AUTO	77.61 ± 0.43%	0.66 ± 0.01	-
		Excluded	53.03 ± 0.60%	0.39 ± 0.01	-
	IHC	AUTO	-	-	53.80 ± 0.39%
		Excluded	-	-	34.90 ± 0.41%
ER- (N = 64)	NCN-PAM50	AUTO	87.84 ± 0.33%	0.79 ± 0.01	-
		Excluded	62.16 ± 0.36%	0.45 ± 0.00	-
	IHC	AUTO	-	-	69.88 ± 0.27%
		Excluded	-	-	53.30 ± 0.33%
TN (N = 64)	NCN-PAM50	AUTO	88.35 ± 0.33%	0.75 ± 0.01	-
		Excluded	56.74 ± 0.56%	0.32 ± 0.01	-
	IHC	AUTO	-	-	69.85 ± 0.27%
		Excluded	-	-	47.00 ± 0.45%

6.3.2 ABiM100 and OSLO2-EMIT0

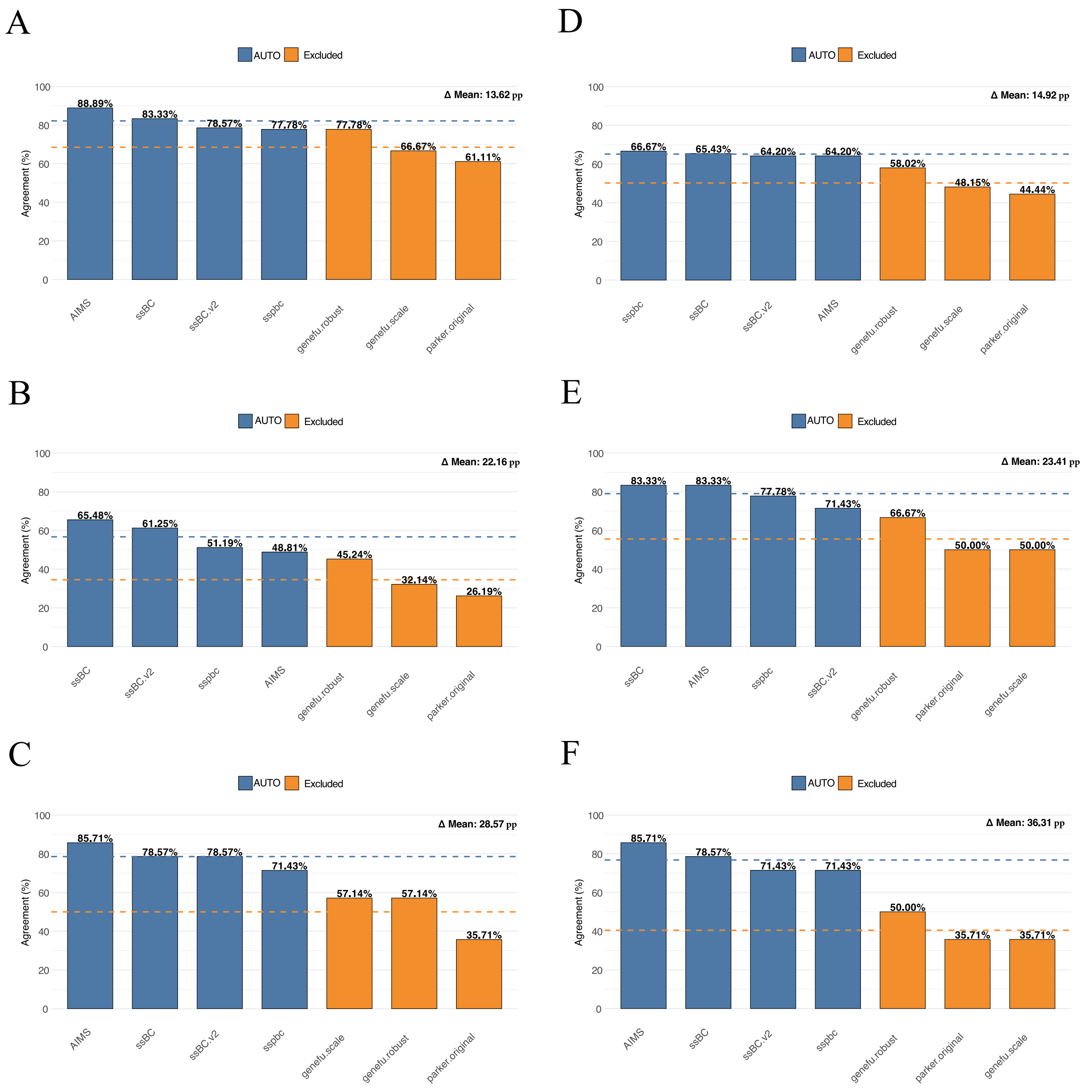

Figure S5. Performance comparison of AUTO (blue) versus Excluded approaches (orange) in agreement with IHC-based subtypes in the ABiM100 and OSLO2-EMIT0 cohorts. Bar plots (A-C) show agreement rates in OSLO2-EMIT0 for (A) pure ER+ (N = 84), (B) ER- (N = 18), and (C) triple-negative (TN; N = 14) subcohorts. Bar plots (D-F) show agreement rates in ABiM100 for (D) pure ER+ (N = 84), (E) ER- (N = 18), and (F) TN (N = 14) subcohorts. Horizontal dashed lines indicate group means, and the mean difference (Δ) between the AUTO and Excluded methods is annotated in each panel. pp: percentage points.

6.3.3 Entropy versus Overall Accuracy

To assess the relationship between Shannon entropy and subtyping accuracy, we evaluated per-sample overall accuracy, defined as NCN-PAM50 concordance rate, across intrinsic molecular subtyping methods in the SCAN-B cohort (N = 4,606) using AUTO mode. The NCN-PAM50 concordance rate represents the fraction of classifiers that agree with the NCN-PAM50 reference for each sample. Entropy was calculated based on the distribution of IS predictions per sample, with lower values indicating higher inter-method consensus. Spearman correlation (ρ) between entropy and NCN-PAM50 concordance was computed using *corr.test* function in psych v2.5.3 R package (27). Samples were further grouped into discrete bins of entropy. The mean concordance rate and SE for each bin were calculated.

While entropy reflects inter-method concordance rather than ground-truth accuracy, it exhibits a strong inverse association with prediction concordance rate ($\rho = -0.99$, $p < 0.001$). Figure S6 displays the distribution of NCN-PAM50 concordance rates within each entropy bin. Boxplots represent the median and interquartile range (IQR), while red diamonds indicate the mean concordance per bin. Samples with lower entropy exhibited higher overall accuracy, demonstrating that AUTO not only improves classification agreement but also minimises inter-classifier discordance. In contrast, samples with higher entropy showed greater variability and reduced concordance, underscoring the importance of assumption-aware method selection in heterogeneous cohorts.

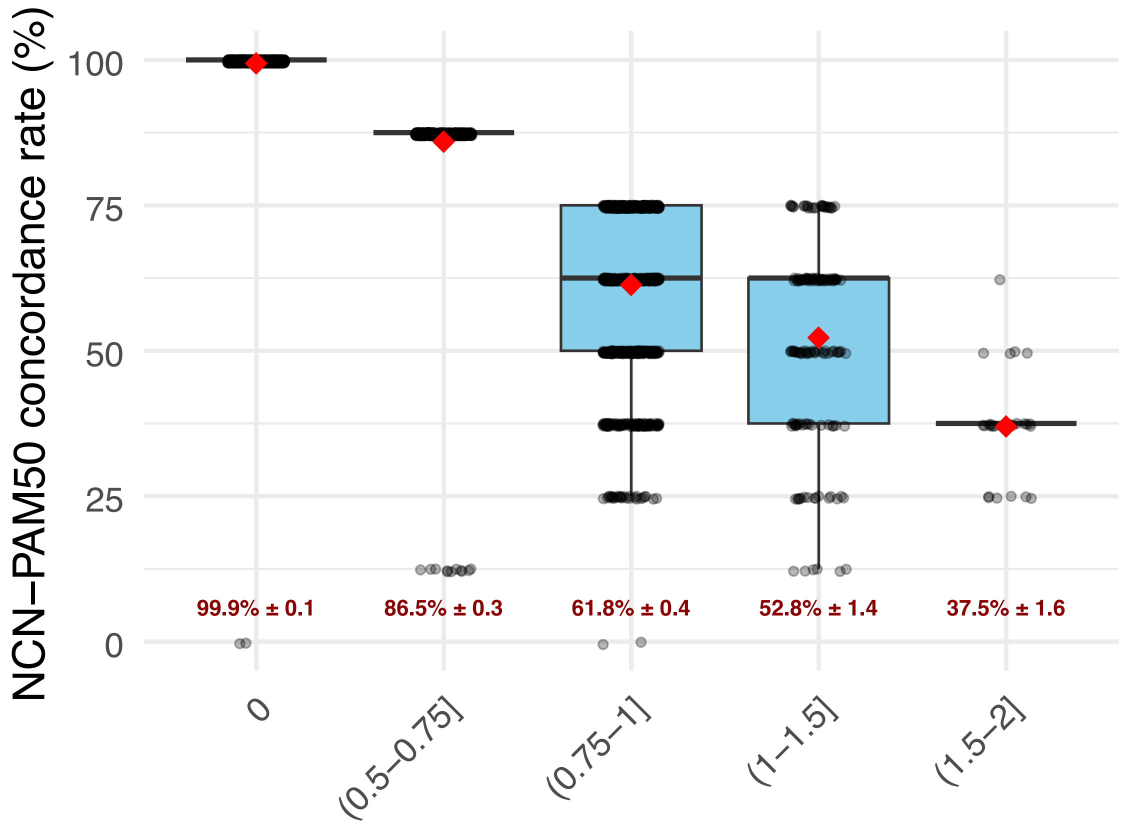

Figure S6. Distribution of NCN-PAM50 concordance rates across entropy bins in the SCAN-B cohort (N = 4,606). Entropy was calculated per sample based on the distribution of IS predictions using AUTO-selected subtyping methods. The NCN-PAM50 concordance rate for each sample (%) was defined as the fraction of methods that agreed with the NCN-PAM50 reference. Entropy values were grouped into eight discrete bins: 0 (perfect agreement), (0–0.25], (0.25–0.5], (0.5–0.75], (0.75–1], (1–1.5], (1.5–2], and ≥2. Boxplots display the median and interquartile range (IQR), while red diamonds indicate the mean concordance rate within each bin. The mean concordance rate and SE were annotated for each bin in red text.

6.3.4 SSP Robustness to ER+ Skew

To evaluate the robustness of SSP-based methods to cohort composition, we compared AIMS and the original PAM50 implementation by Parker et al. 2009 (parker.original) across SCAN-B subcohorts with varying ER+ prevalence. Using NCN-PAM50 as the reference, we assessed

classification performance in balanced (ER+ ≈ 45.8%), low-ER+ (S10; ER+ ≈ 10%), and high-ER+ (S90; ER+ ≈ 90%) settings. AIMS consistently maintained stable accuracy and error rates across all scenarios, while Parker et al. showed marked performance degradation under skewed conditions. These findings support the assumption-independence of SSP-based methods and their suitability for heterogeneous or biased cohorts.

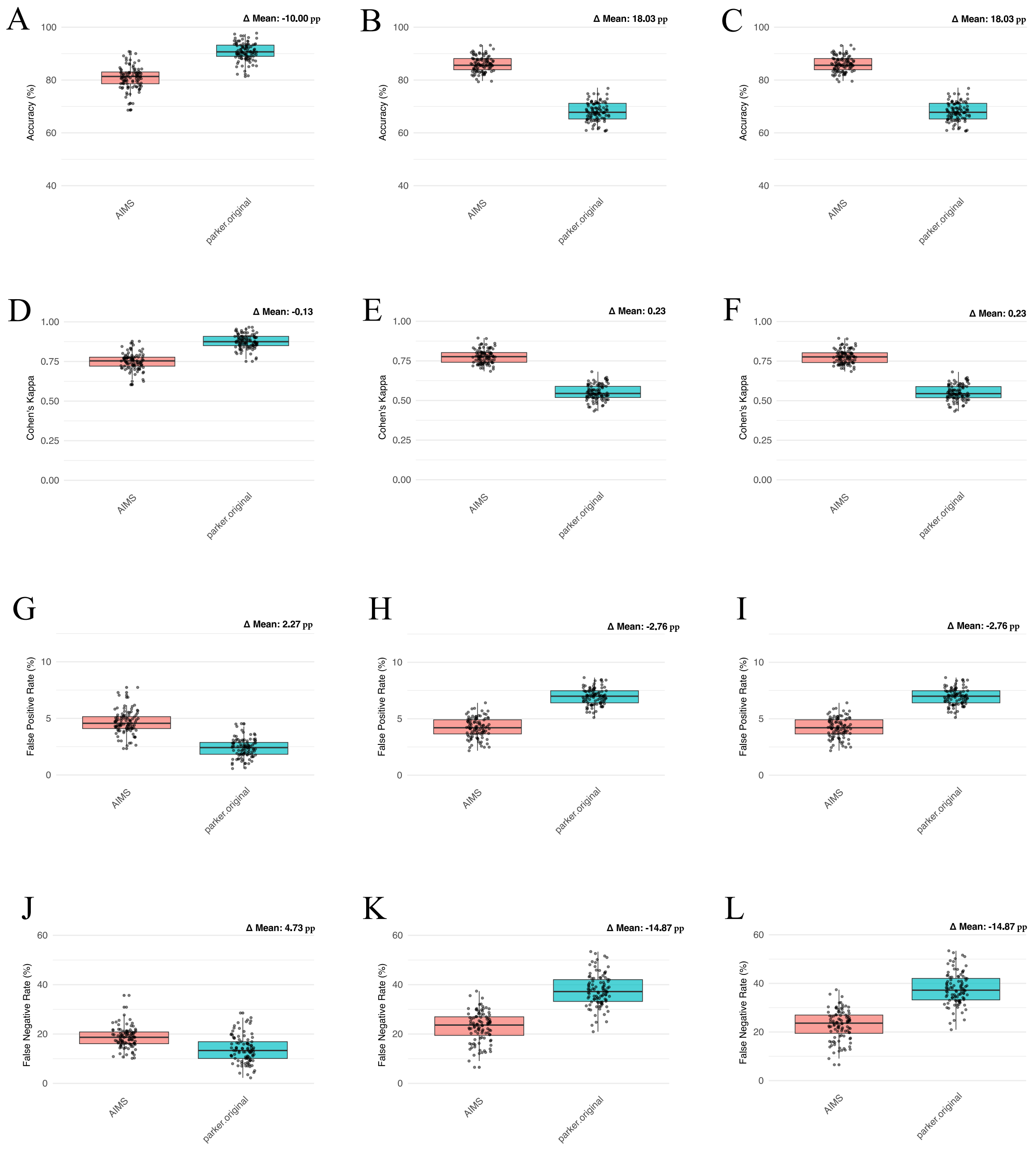

Figure S7. Performance comparison between AIMS (red) and Parker et al 2009 PAM50 (parker.original; blue) against NCN-PAM50 in SCAN-B subcohorts with varying ER+ prevalence. Bar plots show overall accuracy distributions for (A) balanced ER+ (ER+ ≈ 45.8%), (B) low-ER+ (S10; ER+ ≈ 10%), and (C) high-ER+ (S90; ER+ ≈ 90%) subcohorts. Cohen's kappa distributions are shown in (D-F), false positive rates (FPR; %) in (G-I), and false negative rates (FNR; %) in (J-L). Each distribution is based on 100 random subcohorts (N = 118). pp: percentage points.

Table S6. Performance metrics (mean ± SE) comparing AIMS and Parker et al. 2009 PAM50 against NCN-PAM50 in SCAN-B subcohorts with varying ER+ prevalence (ER+ ≈ 45.8%, 10%, 90%). Metrics include accuracy, Cohen's kappa, false positive rate (FPR), and false negative rate (FNR), averaged across 100 replicates per condition.

Subcohort	Method	Accuracy (Mean ± SE)	Kappa (Mean ± SE)	FPR (Mean ± SE)	FNR (Mean ± SE)
Balanced (ER+ ≈ 45.8%)	AIMS	80.72 ± 0.41%	0.7495 ± 0.0051	4.64 ± 0.10%	18.6 ± 0.42%
	parker.original	90.72 ± 0.33%	0.8763 ± 0.0043	2.37 ± 0.08%	13.87 ± 0.55%
S10 (ER+ ≈ 10%)	AIMS	86.05 ± 0.29%	0.7776 ± 0.0044	4.2 ± 0.09%	22.89 ± 0.61%
	parker.original	68.03 ± 0.34%	0.5506 ± 0.0049	6.96 ± 0.07%	37.75 ± 0.65%
S90 (ER+ ≈ 90%)	AIMS	73.81 ± 0.40%	0.6278 ± 0.0055	6.66 ± 0.11%	18.49 ± 42.19%
	parker.original	61.07 ± 0.54%	0.5015 ± 0.0066	8.7 ± 0.12%	22.46 ± 36.22%

7 Usage of BreastSubtypeR

This section provides a step-by-step guide to using the BreastSubtypeR package, highlighting its key features, including the dynamic AUTO mode for method selection, the method-specific normalization pipeline and optimized gene mapping strategy, and the iBreastSubtypeR Shiny application.

7.1 Input Data Preprocessing and Requirements

BreastSubtypeR requires gene expression data to be provided as a *SummarizedExperiment* object. Below, we include comprehensive R pseudocode to demonstrate how various input data types can be processed, including raw RNA-seq count data, normalized RNA-seq data, and normalized microarray/nCounter data. The code clearly specifies the expected data format for both NC-based and SSP-based classification categories.

a. Raw RNA-seq counts (with gene lengths)

```
# NC-based methods: log2-CPM (upper-quartile normalized)
process_nc_counts <- function(counts) {
  lib.size.factors <- edgeR::calcNormFactors(counts, method = "upperquartile")
  log2_uqCPM <- edgeR::cpm(counts,
                        normalized.lib.sizes = TRUE,
                        log = TRUE,
                        prior.count = 1)
  return(log2_uqCPM)
}
log2_uqCPM <- process_nc_counts(raw_counts)   # NC methods

# SSP-based methods: FPKM (linear scale)
fpkm_raw   <- edgeR::rpkm(counts, gene.length = gene.lengths)
```

b. Precomputed RNA-seq FPKM (log$_2$-transformed)

```
# NC-based: Use as-is
nc_matrix <- user_log2fpkm_matrix

# SSP-based: Convert back to linear FPKM
ssp_matrix <- 2^user_log2fpkm_matrix  # Reverse log₂(FPKM)
```

c. Normalized microarray or nCounter expression (log$_2$-transformed)

```
# NC-based: Use as-is
nc_matrix <- user_microarray_matrix

# SSP-based: Convert to linear scale
ssp_matrix <- 2^user_microarray_matrix  # Simple exponentiation
```

The package supports three analysis strategies with specific data requirements.

a. Single-Method Subtyping

- NC-based methods (e.g., parker.original, ssBC, cIHC):
  Log$_2$-transformed expression matrix (gene symbols × samples) in assay
  Clinical information required for cIHC, cIHC.itr, ssBC, ssBC.v2, and PCAPAM50
- SSP-based methods (e.g., AIMS, sspbc):
  Non-log-transformed matrix (Entrez IDs × samples) in assay
  Clinical information optional

b. Multi-Method Subtyping

- The initial Mapping function requires:
  Log$_2$-transformed expression (probes/genes × samples) in assay
  Probe annotation (probe + ENTREZID columns) in rowData
  Clinical metadata in colData

- Output feeds directly into BS_Multi function

c. iBreastSubtypeR Shiny Application

- Accepts text/CSV files matching the Mapping function's structure
- Upload formats: expression matrix, annotation file, clinical data (Fig. S9)

7.2 Output

1. Single-Method Subtyping Results:

   For each method, the output consists of a structured list that includes:

- A table of predicted IS (e.g., Luminal A, Luminal B, HER2-enriched) for each sample.
- Original method outputs, such as correlation values and genomic risk scores for NC-based methods and posterior probabilities for the AIMS method (9).

2. Multiple-Method Subtyping Results:

- The *BS_Multi* function integrates predicted IS for each sample across selected methods (either using AUTO mode or manual selection).
- Shannon entropy is provided for each sample as a supplementary metric to assess concordance between methods.
- The original outputs from each subtyping method are available for further analysis and interpretation.

3. iBreastSubtypeR Shiny Application Results:

   The output includes both visualization and downloadable files. For single-method subtyping, the visualizations consist of a pie chart depicting the distribution of IS within the cohort and a heatmap illustrating clustering of relevant genes (Fig. S9). For multiple-method subtyping (AUTO mode), the visualization is a heatmap similar to that shown in Fig. S8. Additionally, results can be downloaded as text (.txt) files for downstream analysis.

7.3 A case study: The OSLO2-EMIT0 Cohort

Below, we demonstrate how to use AUTO mode and the iBreastSubtypeR Shiny application for performing subtyping analysis, using OSLO2-EMIT0 (20,21).

7.3.1 Performing Multi-Method Intrinsic Subtyping Using AUTO Mode

```
## Install the package (if not already installed)
# Install the released version from Bioconductor:
if (!require("BiocManager", quietly = TRUE)) install.packages("BiocManager")
BiocManager::install("BreastSubtypeR")

# Or install from GitHub:
# Install remotes package if you haven't already
if (!require("remotes", quietly = TRUE)) install.packages("remotes")
# Install BreastSubtypeR from GitHub
remotes::install_github("JohanHartmanGroupBioteam/BreastSubtypeR")

# Load the package
library(BreastSubtypeR)

# Load example data
data("BreastSubtypeRobj")
data("OSLO2EMIT0obj")

# Perform method-specific pre-processing and gene mapping before subtyping
data_input <- Mapping( OSLO2EMIT0obj$se_obj, RawCounts = FALSE, impute = TRUE, verbose = TRUE )

# Perform multi-method subtyping using AUTO mode
result <- BS_Multi(
  data_input = data_input,
  methods = "AUTO",
  Subtype = FALSE,
  hasClinical = FALSE
)

# View the subtyping results
head(result$res_subtypes)

# Visualize results
plot <- Vis_Multi(result$res_subtypes)
plot(plot)
```

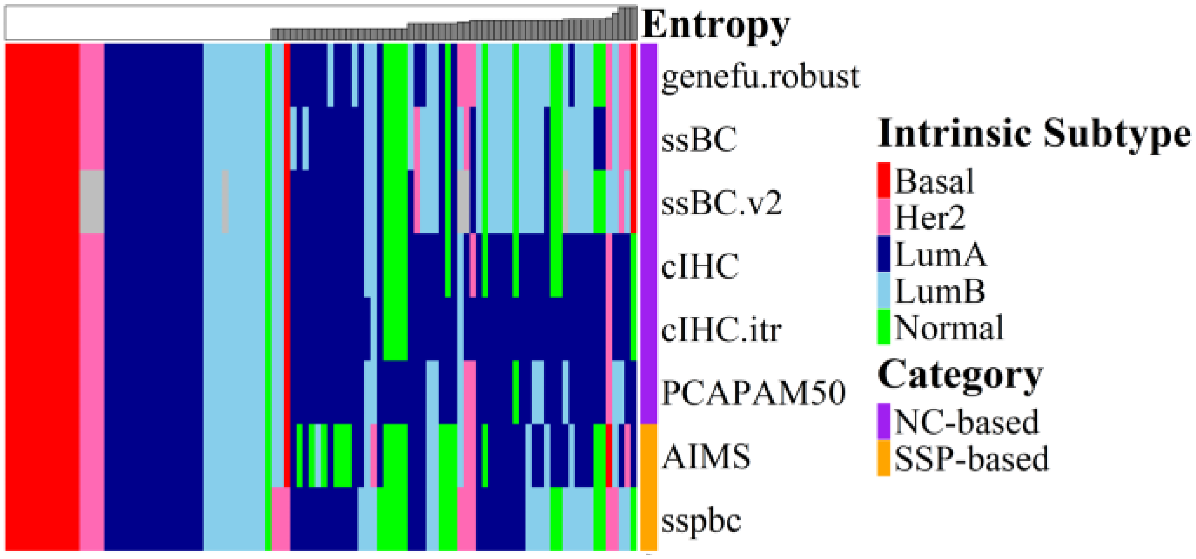

Figure S8. Visualization of Multi-Method Subtyping Results from BreastSubtypeR. Example visualization output from the BreastSubtypeR package using the *Vis_Multi* function after running *BS_Multi* in AUTO mode for the OSLO2-EMIT0 cohort. Each row represents a subtyping method, and each column corresponds to a patient sample. Patient samples are sorted by entropy value (from smallest to largest, left to right) and then by IS from the last method (sspbc). Gray indicates NA values. NC-based: nearest centroid-based methods. SSP-based: single-sample predictor methods. LumA: Luminal A, LumB: Luminal B, Her2: HER2-enriched, Basal: Basal-like, Normal: Normal-like.

7.3.2 Performing Single-Method Intrinsic Molecular Subtyping Using the iBreastSubtypeR Shiny Application

```
# Install the released version from Bioconductor:
if (!require("BiocManager", quietly = TRUE)) install.packages("BiocManager")
BiocManager::install("BreastSubtypeR")

# Or install from GitHub:
# Install remotes package if you haven't already
if (!require("remotes", quietly = TRUE)) install.packages("remotes")
# Install BreastSubtypeR from GitHub
remotes::install_github("JohanHartmanGroupBioteam/BreastSubtypeR")

# Launch the local Shiny app for interactive analysis
# The example files are located under inst/RshinyTest on GitHub.
BreastSubtypeR::iBreastSubtypeR() # interactive GUI (local)
```

Interactive Breast Cancer Intrinsic Molecular Subtyping

Welcome to iBreastSubtypeR

An interactive companion to the BreastSubtypeR Bioconductor package for intrinsic molecular subtyping.

NC-based: PAM50 (parker.original | genefu.scale | genefu.robust), cIHC / cIHC.itr, PCAPAM50, ssBC / ssBC.v2 · **SSP-based:** AIMS, SSPBC · **ROR:** research-use Risk of Recurrence (uses TSIZE, NODE; NC methods only)

AUTO: assumption-aware selection — action needed · Why AUTO?

- Assumption-aware AUTO reduces bias in ER/HER2-skewed or subtype-specific cohorts.
- Standardised mapping & normalisation across platforms (log_2-CPM for NC; FPKM for SSP).
- Choose 5-class (incl. Normal-like) or 4-class; AIMS is 5-class only.
- All computation runs locally; exports are Bioconductor-ready.

Please cite: Yang Q., Hartman J., Sifakis E.G. *BreastSubtypeR: A Unified R/Bioconductor Package for Intrinsic Molecular Subtyping in Breast Cancer Research*. NAR Genomics and Bioinformatics (2025). https://doi.org/10.1093/nargab/lqaf131

Go to Step 1 | Go to Step 2 | About & Citation

Step 1 · Upload your data

1) Gene expression (GEX)

Upload expression matrix

Browse... OSLO2EMIT0_GEX_lc

Upload complete

Data type

(●) Normalized (log2) ( ) Raw counts (RNA-seq)

Expression matrix requirements Selected: Normalized (log2)
- **Format:** genes × samples *log2-normalized expression* .
- **Examples:** `log2(FPKM+1)` , microarray/nCounter log2.
- **Rows:** gene IDs matching `annotation$probe` .
- **Columns:** sample IDs matching `clinical$PatientID` .
- **Processing:** NC → uses log2 values as-is; SSP → back-transforms to linear scale.

2) Clinical data

Upload clinical table

Browse... OSLO2EMIT0_clinica

Upload complete

Clinical data requirements
- **Minimum:** `PatientID` .
- **Method-specific:** cIHC / cIHC.itr / PCAPAM50 need `ER` (values `ER+` / `ER-`). ssBC requires `ER` (and `HER2` for `ER.v2`) or `TN` depending on subgroup.
- **Optional for ROR (NC methods):** include `TSIZE` and `NODE` .

3) Feature annotation

Upload annotation table

Browse... OSLO2EMIT0_anno.t

Upload complete

Feature annotation requirements
- `probe` — matches GEX row names.
- `ENTREZID` — mandatory.

Preprocess & map

Runs Mapping() to align IDs and normalize (as needed). When complete, continue to Step 2.

Step 2 · Choose method & parameters

Subtyping method

PAM50 (parker.original | genefu.scale | genefu.robust)

Subtype classes

(●) 5 classes (includes Normal-like) ( ) 4 classes (excludes Normal-like)

PAM50 family (NC-based)
- **Variants:** parker.original | genefu.scale | genefu.robust
- **IHC Input Requirement:** Not required
- **Description:** Nearest-centroid subtyping using the PAM50 gene set; multiple centering options.
- **Key refs:** Parker et al., 2009 JCO (doi); Gendoo et al., 2016 Bioinformatics (doi)

[ ] Use clinical variables (ROR)

Calibration strategy: Internal

Internal calibration method: medianCtr (parker.original)

Calibration notes (PAM50)
- **None**: no centering (use only if your expression scale already matches the training data).
- **Internal**: center your cohort; choose one of:
  - `medianCtr` — gene-wise *median* centering (Parker original).
  - `meanCtr` — gene-wise z-score (mean 0, sd 1).
  - `qCtr` — robust quantile re-centering (mq ≈ 0.05).
- **External**: subtract *reference medians* from a training cohort/platform.
  - **Reference medians** selector: choose a *Built-in* preset (e.g., `RNAseq.V2` , `nCounter` , `Agilent_244K`) or **Custom (upload file...)**.
  - **Custom upload**: CSV/TXT with two columns — `X` (PAM50 gene symbol) and `Given.mdns` (median log2 expression). Exactly 50 unique symbols; extra rows are ignored; gene symbols are case-sensitive.
- **Gene overlap**: all steps operate on the intersection of your genes and PAM50; missing genes are ignored.

Run subtyping

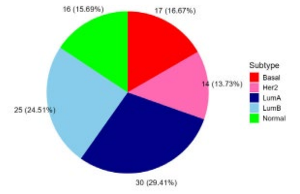

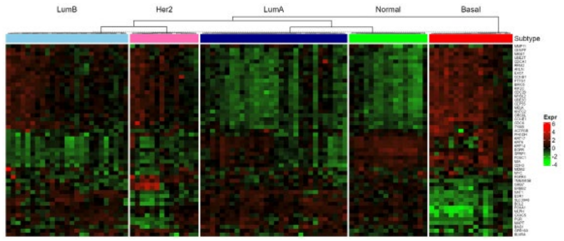

Export content

(●) Calls only ( ) Full metrics (incl. ROR when available)

Download results (.tsv)

© Karolinska Institutet — BreastSubtypeR. DOI: 10.1093/nargab/lqaf131

Figure S9. Visualization of Single-Method Subtyping in the iBreastSubtypeR Shiny Application. The screenshot shows the two-step interactive workflow. **Step 1**: The user uploads (i) a gene expression file (Input 1; log2-normalized RNA-seq/microarray/nCounter, or raw RNA-seq counts; non-gene-centered matrix with rows as probes and columns as samples), (ii) a clinical information file (Input 2; patient ID, clinical attributes), and (iii) a probe annotation file (Input 3; probe and ENTREZID columns, plus Length for raw counts). **Step 2**: The user selects a subtyping method and its parameters. The output includes visualizations and downloadable (.txt) files for downstream analysis.

8 Statistical Analysis and Visualization

All visualizations were generated using functions from the BreastSubtypeR v1.1.3 and ggplot2 v4.0.0 R packages (28). Statistical analyses and visualizations were performed entirely within R version 4.5.0.

Reference

1. Harbeck N, Penault-Llorca F, Cortes J, Gnant M, Houssami N, Poortmans P, et al. Breast cancer. Nat Rev Dis Primers [Internet]. 2019;5(1):66. Available from: https://doi.org/10.1038/s41572-019-0111-2
2. Koboldt DC, Fulton RS, McLellan MD, Schmidt H, Kalicki-Veizer J, McMichael JF, et al. Comprehensive molecular portraits of human breast tumours. Nature [Internet]. 2012;490(7418):61–70. Available from: https://doi.org/10.1038/nature11412
3. Sørlie T, Perou CM, Tibshirani R, Aas T, Geisler S, Johnsen H, et al. Gene expression patterns of breast carcinomas distinguish tumor subclasses with clinical implications. Proc Natl Acad Sci U S A. 2001;98(19).
4. Perou CM, Sørlie T, Eisen MB, van de Rijn M, Jeffrey SS, Rees CA, et al. Molecular portraits of human breast tumours. Nature [Internet]. 2000;406(6797):747–52. Available from: https://doi.org/10.1038/35021093
5. Wallden B, Storhoff J, Nielsen T, Dowidar N, Schaper C, Ferree S, et al. Development and verification of the PAM50-based Prosigna breast cancer gene signature assay. BMC Med Genomics. 2015;8(1).
6. Nielsen T, Wallden B, Schaper C, Ferree S, Liu S, Gao D, et al. Analytical validation of the PAM50-based Prosigna Breast Cancer Prognostic Gene Signature Assay and nCounter Analysis System using formalin-fixed paraffin-embedded breast tumor specimens. BMC Cancer. 2014;14(1).
7. Prat A, Parker JS. Standardized versus research-based PAM50 intrinsic subtyping of breast cancer. Vol. 22, Clinical and Translational Oncology. 2020.
8. Parker JS, Mullins M, Cheang MCU, Leung S, Voduc D, Vickery T, et al. Supervised Risk Predictor of Breast Cancer Based on Intrinsic Subtypes. Journal of Clinical Oncology [Internet]. 2009 Feb 9;27(8):1160–7. Available from: https://doi.org/10.1200/JCO.2008.18.1370
9. Paquet ER, Hallett MT. Absolute assignment of breast cancer intrinsic molecular subtype. J Natl Cancer Inst. 2015;107(1).
10. Staaf J, Ringnér M. Making breast cancer molecular subtypes robust? Vol. 107, Journal of the National Cancer Institute. 2015.

11. Schettini F, Brasó-Maristany F, Kuderer NM, Prat A. A perspective on the development and lack of interchangeability of the breast cancer intrinsic subtypes. Vol. 8, npj Breast Cancer. 2022.
12. Gendoo DMA, Ratanasirigulchai N, Schröder MS, Paré L, Parker JS, Prat A, et al. Genefu: An R/Bioconductor package for computation of gene expression-based signatures in breast cancer. Bioinformatics. 2016;32(7).
13. Zhao X, Rødland EA, Tibshirani R, Plevritis S. Molecular subtyping for clinically defined breast cancer subgroups. Breast Cancer Research. 2015;17(1).
14. Fernandez-Martinez A, Krop IE, Hillman DW, Polley MY, Parker JS, Huebner L, et al. Survival, pathologic response, and genomics in CALGB 40601 (Alliance), a neoadjuvant Phase III trial of paclitaxel-trastuzumab with or without lapatinib in HER2-positive breast cancer. In: Journal of Clinical Oncology. 2020.
15. Curtis C, Shah SP, Chin SF, Turashvili G, Rueda OM, Dunning MJ, et al. The genomic and transcriptomic architecture of 2,000 breast tumours reveals novel subgroups. Nature. 2012;486(7403).
16. Ciriello G, Gatza ML, Beck AH, Wilkerson MD, Rhie SK, Pastore A, et al. Comprehensive Molecular Portraits of Invasive Lobular Breast Cancer. Cell. 2015;163(2).
17. Staaf J, Häkkinen J, Hegardt C, Saal LH, Kimbung S, Hedenfalk I, et al. RNA sequencing-based single sample predictors of molecular subtype and risk of recurrence for clinical assessment of early-stage breast cancer. NPJ Breast Cancer. 2022;8(1).
18. Raj-Kumar PK, Liu J, Hooke JA, Kovatich AJ, Kvecher L, Shriver CD, et al. PCA-PAM50 improves consistency between breast cancer intrinsic and clinical subtyping reclassifying a subset of luminal A tumors as luminal B. Sci Rep. 2019;9(1).
19. Winter C, Nilsson MP, Olsson E, George AM, Chen Y, Kvist A, et al. Targeted sequencing of BRCA1 and BRCA2 across a large unselected breast cancer cohort suggests that one-third of mutations are somatic. Annals of Oncology. 2016;27(8).
20. Aure MR, Vitelli V, Jernström S, Kumar S, Krohn M, Due EU, et al. Integrative clustering reveals a novel split in the luminal A subtype of breast cancer with impact on outcome. Breast Cancer Research. 2017;19(1).

21. Tekpli X, Lien T, Røssevold AH, Nebdal D, Borgen E, Ohnstad HO, et al. An independent poor-prognosis subtype of breast cancer defined by a distinct tumor immune microenvironment. Nat Commun. 2019;10(1).
22. Goldhirsch A, Wood WC, Coates AS, Gelber RD, Thürlimann B, Senn HJ. Strategies for subtypes-dealing with the diversity of breast cancer: Highlights of the St Gallen international expert consensus on the primary therapy of early breast cancer 2011. Annals of Oncology. 2011;22(8).
23. Montagna E, Bagnardi V, Rotmensz N, Viale G, Cancello G, Mazza M, et al. Immunohistochemically defined subtypes and outcome in occult breast carcinoma with axillary presentation. Vol. 129, Breast Cancer Research and Treatment. 2011.
24. Goldhirsch A, Winer EP, Coates AS, Gelber RD, Piccart-Gebhart M, Thürlimann B, et al. Personalizing the treatment of women with early breast cancer: Highlights of the st gallen international expert consensus on the primary therapy of early breast Cancer 2013. Annals of Oncology. 2013;24(9).
25. Brueffer C, Vallon-Christersson J, Grabau D, Ehinger A, Häkkinen J, Hegardt C, et al. Clinical Value of RNA Sequencing–Based Classifiers for Prediction of the Five Conventional Breast Cancer Biomarkers: A Report From the Population-Based Multicenter Sweden Cancerome Analysis Network—Breast Initiative. JCO Precis Oncol. 2018;(2).
26. Leek JT, Johnson WE, Parker HS, Jaffe AE, Storey JD. The SVA package for removing batch effects and other unwanted variation in high-throughput experiments. Bioinformatics. 2012;28(6).
27. Revelle W. Package "psych" - Procedures for Psychological, Psychometric and Personality Research. R Package version 2.5.3. 2015.
28. Wilkinson L. ggplot2: Elegant Graphics for Data Analysis by WICKHAM, H. Biometrics. 2011;67(2).
